# siRNA as a criterion in host immunity and cancer immunotherapy: modulating factors and nano-conjugate based approach for intervention

**DOI:** 10.7150/ijbs.109637

**Published:** 2025-07-28

**Authors:** Rahul Bhattacharjee, Debanjan Das, Srija Chakraborty, Radheka Bhaduri, Soham Chattopadhyay, Rajeev K Singla, Vinoth Kumarasamy, Rohit Gundamaraju

**Affiliations:** 1 KIIT School of Biotechnology, Kalinga Institute of Industrial Technology (KIIT-DU), Bhubaneswar, Odisha, India.; 2 St Xavier's College (Autonomous), Kolkata, West Bengal, India.; 3 Department of Zoology, Maulana Azad College, Kolkata, Kolkata-700013, West Bengal, India.; 4 Department of Parasitology and Medical Entomology, Faculty of Medicine, Universiti Kebangsaan Malaysia, Jalan Yaacob Latif, 56000, Kuala Lumpur, Malaysia.; 5 ER stress and Mucosal immunology lab, School of Health Sciences, University of Tasmania, Launceston, Tasmania, Australia.; 6 Department of Pharmaceutical Engineering, BV Raju Institute of Technology, Narsapur, Medak, Telangana, 502313, India.; 7 Joint Laboratory of Artificial Intelligence for Critical Care Medicine, Department of Critical Care Medicine and Institutes for Systems Genetics, Frontiers Science Center for Disease-related Molecular Network, West China Hospital, Sichuan University, Chengdu, China.; 8 School of Pharmaceutical Sciences, Lovely Professional University, Phagwara, Punjab 144411, India.

**Keywords:** siRNA, nano-conjugates, cancer

## Abstract

The use of Small interfering RNAs (siRNA) is prevalent in various cancer-based therapies. siRNA is a powerful RNAi, which can be used in clinical oncology with nanoparticles as a vector for delivery. A nano-based siRNA conjugated system has been used to target various multi-drug resistance (MDR) genes of cancer to increase therapeutic specificity and control tumor progression using effective delivery. It offers a targeted avenue in gene silencing with reduced off-target effects. Pre-clinical studies show the effectiveness of this combined siRNA-nanoconjugates therapy in chemotherapeutics resistance to cancer cells. This combinatorial approach not only has the potential to induce an immune response inside the host cells but also renders the MDR genes of various cancers ineffective. The current review focuses on the effect of siRNA entry on immune cells and the factors governing them. Moreover, we have further discussed the limiting factor that controls the siRNA-nanoconjugates efficiency for effective tumor regression. We have enumerated the preclinical and clinical significance of this combined therapy for enhanced tumor regression. Furthermore, we have elaborated the impact of this combined nano-conjugated therapy host immune system while pointing out the limitations posed by them. Thus, in essence, this review provides a unique platform for the readers to understand the potential of siRNA-conjugates for anti-cancer therapy from pre-clinical to bench side.

## 1. Introduction

**Figure 1:**
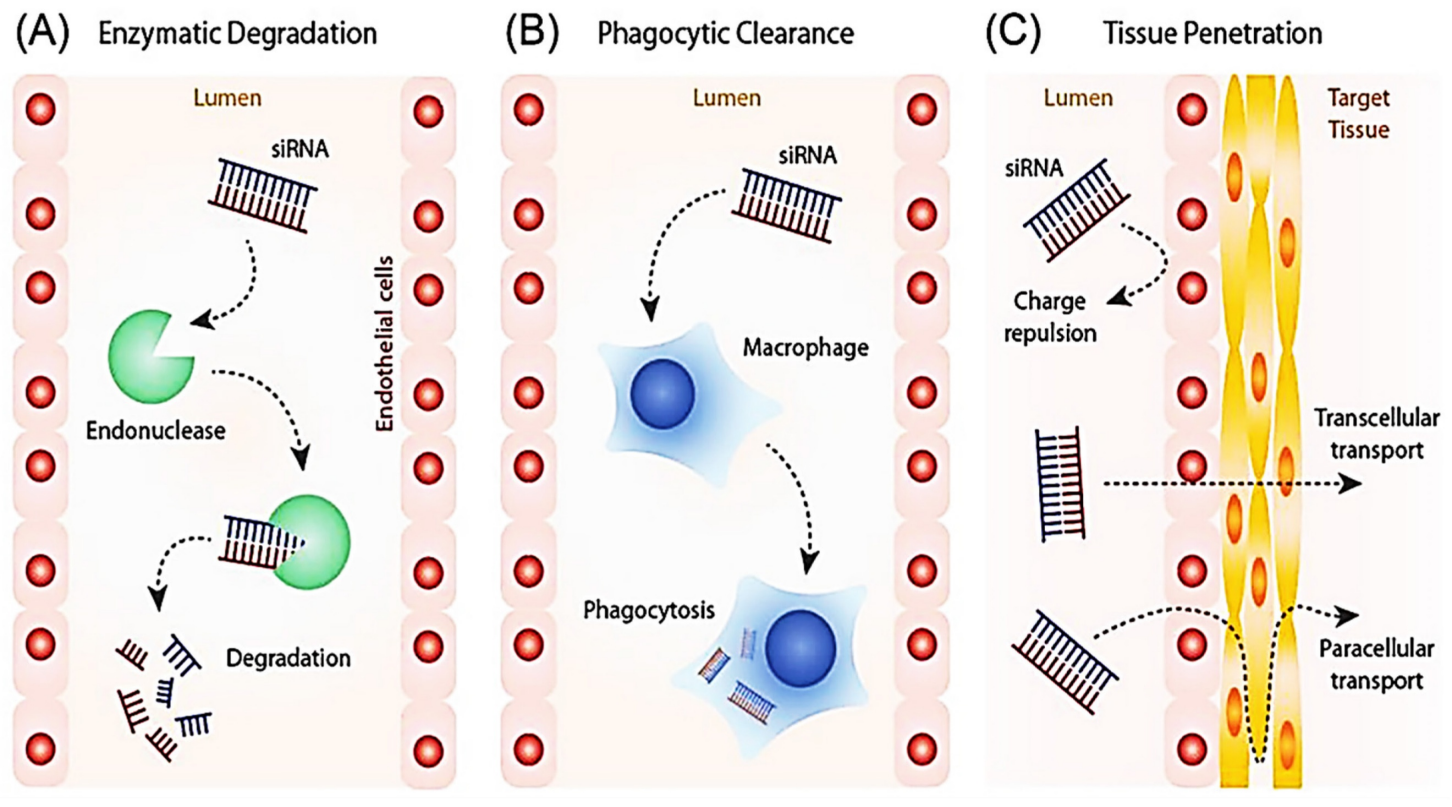
Limitation posed to the delivery of siRNAs in cells. Adapted with open access permission from 19.

Cancer has been accountable for about 8.2 million deaths globally, among which lung cancer is the biggest cause, followed by liver cancer, stomach cancer, and breast cancer 1, 2. Chemotherapy, radiation, and surgery are some of the contemporary cancer treatments. Despite breakthroughs in the fields of surgery and radiotherapy, chemotherapy continues to serve a fundamental role in cancer treatment 3, 4. Chemotherapy, despite being a critical component of cancer treatment, poses several drawbacks, including non-targeted limited delivery of chemotherapeutics leading to failure of drug accumulation and tumor non-responsiveness 5. Multi-resistance, also known as multidrug resistance (MDR), is thought to be the primary source of the penultimate and final limitation 6, 7, 8. A combination of factors like aberrant vasculature, localized hypoxia, low pH environment, up-regulated ABC-transporters, enzymatic degradation, aerobic glycolysis, higher apoptotic threshold, increased interstitial fluid pressure, exosomal miRNAs 8, and a variety of other variables altogether makes up a complex set of mechanisms known as MDR, that diminish the effects of chemotherapy 9-11. The paradigm for cancer treatment is slowly shifting away from non-specific cytotoxic drugs toward selective mechanism-based treatments. Combining immune-targeted gene silencing with other cancer therapies is an untapped option for a better understanding of individual tumor pathways 12, 13. By generating the precise and reversible loss of expression of target genes, RNAi therapies offer the potential to treat a wide range of disorders, including cancer 14, 15. Short interfering RNA (siRNA) has already been shown to influence particular gene expression in cancer cells with tumor regression 16, 17. Thus, we suggest targeting immune cells either separately or in combination. Despite their enormous potential, bare siRNA molecules have several drawbacks, including extremely short half-lives (minutes), poor nuclease protection, low chemical stability, and dissociation from the vectors (Figure 1) 18. As a result, it is critical to explore suitable nanoparticle design and construction for safe and effective siRNA delivery.

siRNA delivery entails the introduction of foreign material into a stable biological environment and hence carries the potential to trigger an immune response 20. For RNAi-mediated treatments, nanoconjugates provide diverse, targeted delivery platforms for targeted delivery while overcoming their current limitations 21-25. Thus, combining RNAi with nanomaterials acts as a powerful weapon to target immune cells for cancer treatment. Combination therapy for cancer treatment has been advocated because of its principal benefit of improved efficacy due to additive or synergistic anti-cancer action 26, 27. With the use of an appropriate combination of chemotherapeutics, a synergistic effect can be achieved, which enhances therapeutic success and patient compliance to lower doses and reduces the development of cancer drug resistance 28, 29.

**Figure 2:**
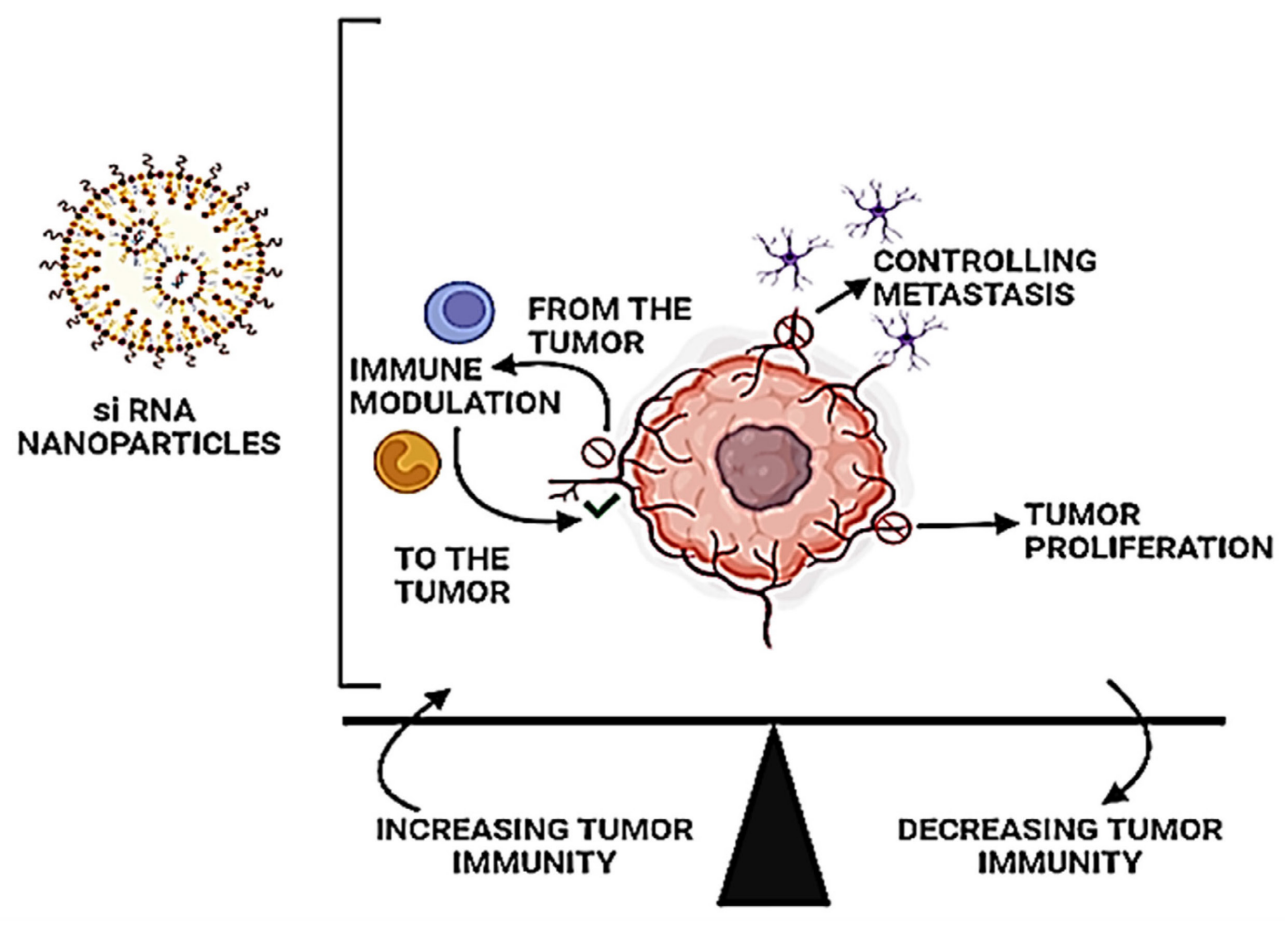
Tumors encourage their growth by creating a diverse environment that suppresses tumor immunity. The use of RNAi nanoparticles to modulate immune responses and restore tumorigenic pathways is a potential new therapeutic approach.

In this review, we are discussing the fundamental functioning of the immune system upon stimulation by the siRNA-nanoparticle-based system and the factors responsible for them (Figure 2). We have not only enumerated the impact of immunomodulation of this combinational therapy on the cancer cells but also how immune stimulation by the siRNA-nanoparticle-based system could activate immune cells to activate against TME. Moreover, we have spread thoughts in both preclinical and clinical studies on the role of combinational therapy for tumor regression against MDR cancer.

## 2. Factors Responsible for Immune Activation via siRNA

Many different properties of siRNA are recognized by the varied repertoire of PRRs (Pattern recognition receptors) found in mammalian systems. This section discusses how siRNA and its associated delivery mechanism can be tailored to stimulate the innate immune system for tumor regression.

### 2.1 Sequence of siRNA

Various strategies have been employed by the innate immune response to identify pathogenic signatures. Toll like receptor-7 (TLR7) recognizes them depending upon their sequence, whereas Toll like receptor (TLR)-independent RNA receptors such as Protein kinase R (PKR) and RIG identify them irrespective of their sequence 30. TLR-7 mediated response was triggered by the presence of a 5'-UGU-3' sequence in RNA 24. It was determined that a sequence rich in GU provoked the immune system to a larger extent while reducing uridine residues led to a contrasting effect. Further studies showed that regardless of the amount of GU nucleosides, the presence of the 5'-GUCCUUCAA-3' motif could lead to immunostimulatory effects, which lead to cytokine production 30. Surprisingly, it has been observed that the sole molecular features required to trigger immune response via TLR7 or TLR8 are the existence of a backbone made of ribose sugar and the presence of numerous U residues in close vicinity with each other 31. These two features set RNA apart from DNA. Thus, all unmodified siRNA triggers an immune reaction to a certain extent, but the amount of it is controlled by the presence of certain sequences in the strand. Hence, changing these specific sequences inside RNA can reduce pro-inflammatory activity, like substituting U for A lowered IFN-ϒ production in pDCs while changing G for A lowered IL-6 and TNF-α production in blood cells 32, 33.

### 2.2 Structure

The structure of the siRNA plays a vital role in the immune response. The uncapped 5'-triphosphate group present in dsRNA or ssRNA; is a feature of viral RNA that triggers an immune response via interferons as RIG-I binds to it 34-36. Furthermore, it was observed that dsRNA, which is blunt-ended, triggers a higher immune response via RIG-I recognition 37. When 3' overhangs are incorporated in both or either RNA, the immune response reduces as RGI loses the capacity to unwind and attach to RNA. From this, it is clear that discrimination between self and non-self RNA has a structural basis. In other words, it can be established that nucleotide structure majorly influences the innate immune system by introducing 2'-O, 4'-C methylene bridge into a ribose ring leading to the formation of locked nucleic acid (LNA) which can effectively reduce immune stimulation caused by the RNA when both the strands of the siRNA were modified 38-42. The potency of the RNAi, however, could change depending on the point of insertion 43. The idea of inserting 2'O-Me was explored in the first place since chemically modifying the 2'-OH group on the sugar ring of the RNA leads to a lesser chance of endonuclease degradation. Unlocked nucleic acids have also been investigated lately as a means of controlling the stability of siRNA duplex and checking on its off-targeting effect. In the initial research, it was observed that adding unlocked nucleic acids (UNA) into the antisense strand prevented off-targeting effects while maintaining the potency of RNAi 44-46. The immunostimulatory capacity of the UNA has not been checked yet, but UNA being a non-viral trait does carry the potential to minimize immunostimulatory reactions.

**Figure 3:**
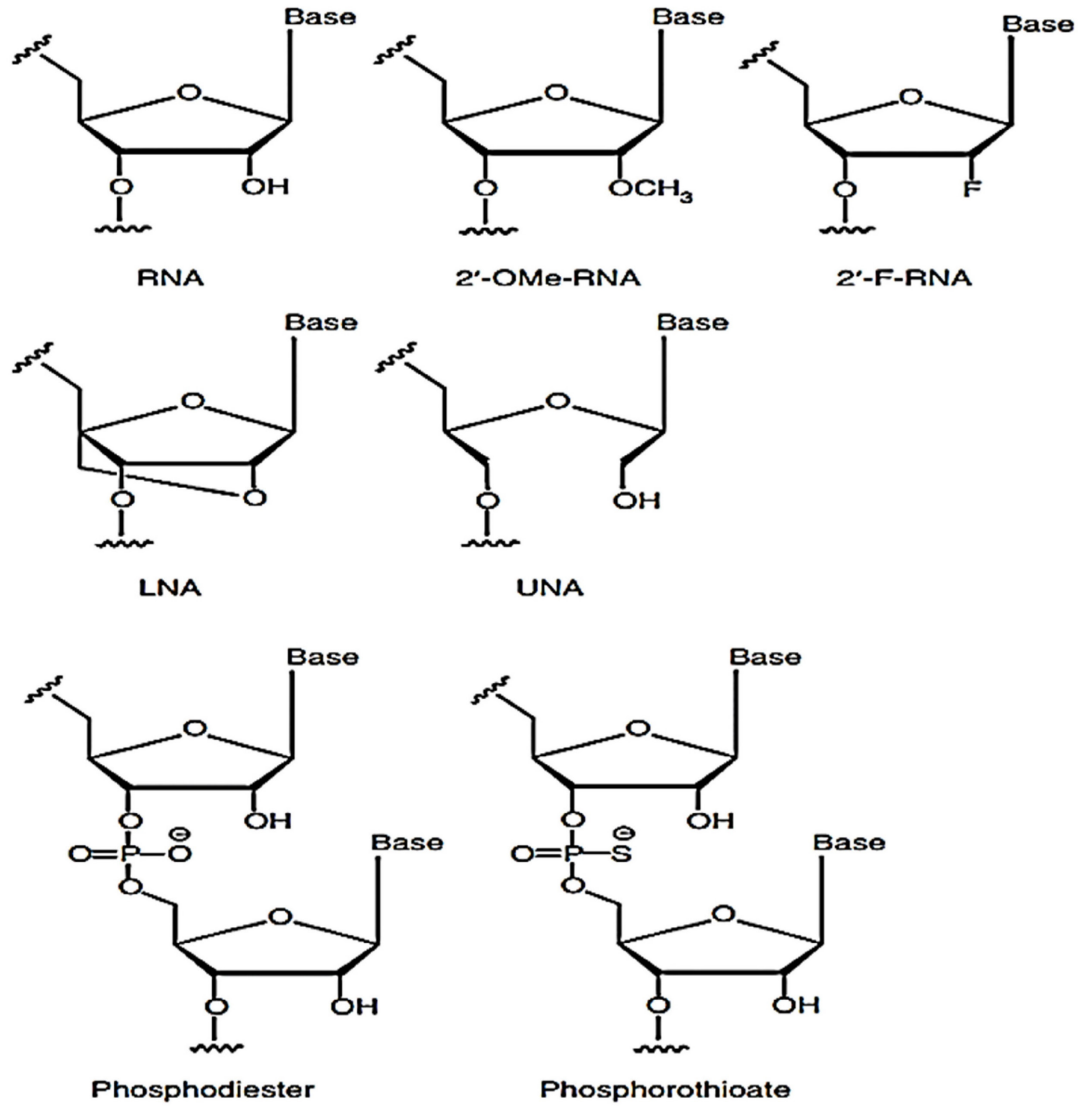
Common chemical changes to the siRNA backbone-Methoxy (2'-OMe) or fluorine (2'-F) moieties can be substituted for the 2'-OH of the ribose ring. A methylene bridge connects the 2' oxygen with the 4' carbon in locked nucleic acids (LNA). There is no linkage between the 2' and 3' carbons in unlocked nucleic acids (UNA). Adapted with open access permission from 20.

Several alternative 2' modifications have been intricately studied by different research groups and are illustrated below (Figure 3). These modifications include 2-O-methyl and 2'-F, 2'-H modifications 43, 47, 48. The number of nucleotides altered in the duplex and the site of insertion determined the extent of immune suppression by 2'F modification 49. The 2'-H alterations, on the other hand, mimic the DNA structure and thus have the capacity to elude immune identification while maintaining normal TLR7/8 activity 50. It was further seen that 2'-H modifications in thymidine or uridine inside RNA strands prevented off-target effects 50. It was further observed that when a siRNA duplex was modified with a mixture of 2'-F modified RNA and DNA analogs leading to improved silencing activity while lowering immune stimulation to a great extent in the blood cells of humans 51.

2'-O-Me modifications seemed to be more effective relative to other modifications since it efficiently suppresses the detection of siRNA via TLR7 or TLR8 and removes RIG-I mediated triggering of the immune system as well 52. When 2-O-Me' modification was introduced into a small number of residues inside the sense siRNA strand, it was able to eliminate immune stimulation without impairing RNAi capacity, which in turn suggested that the ideal method for reducing immune reaction was by carefully introducing 2'-O-Me into both strands of the duplex. However, the changes in the antisense strand should be executed cautiously so that the RNAi activity is not altered 41, 53. 2'-O-Me modification in RNA functions as TLR7 antagonists 42, 54. This antagonistic activity is considered to be a component of self vs. non-self-recognition of the cell as 2'chemical modifications are present on the sugar ring of self RNA. When the TLR7 believes it has identified self-RNA, it stops the further auto-immune reaction. The nucleotide position that is most suitable for this modification was further studied, and it was noted that inserting 2'-O-Me substitution at position 2 of the nucleotide at the antisense strand decreased off-target silencing of mRNA transcripts that had partial complementarity with the antisense strand 55. Modifications of the sense strand at position 9 of nucleotide interfere with RISC assembly and cleavage of the sense strand, hampering the effectiveness of RNAi 49, 56. Thus, the position where the modifications are inserted is crucial 38, 43, 57, 58.

### 2.3 Delivery Vehicle

**Figure 4:**
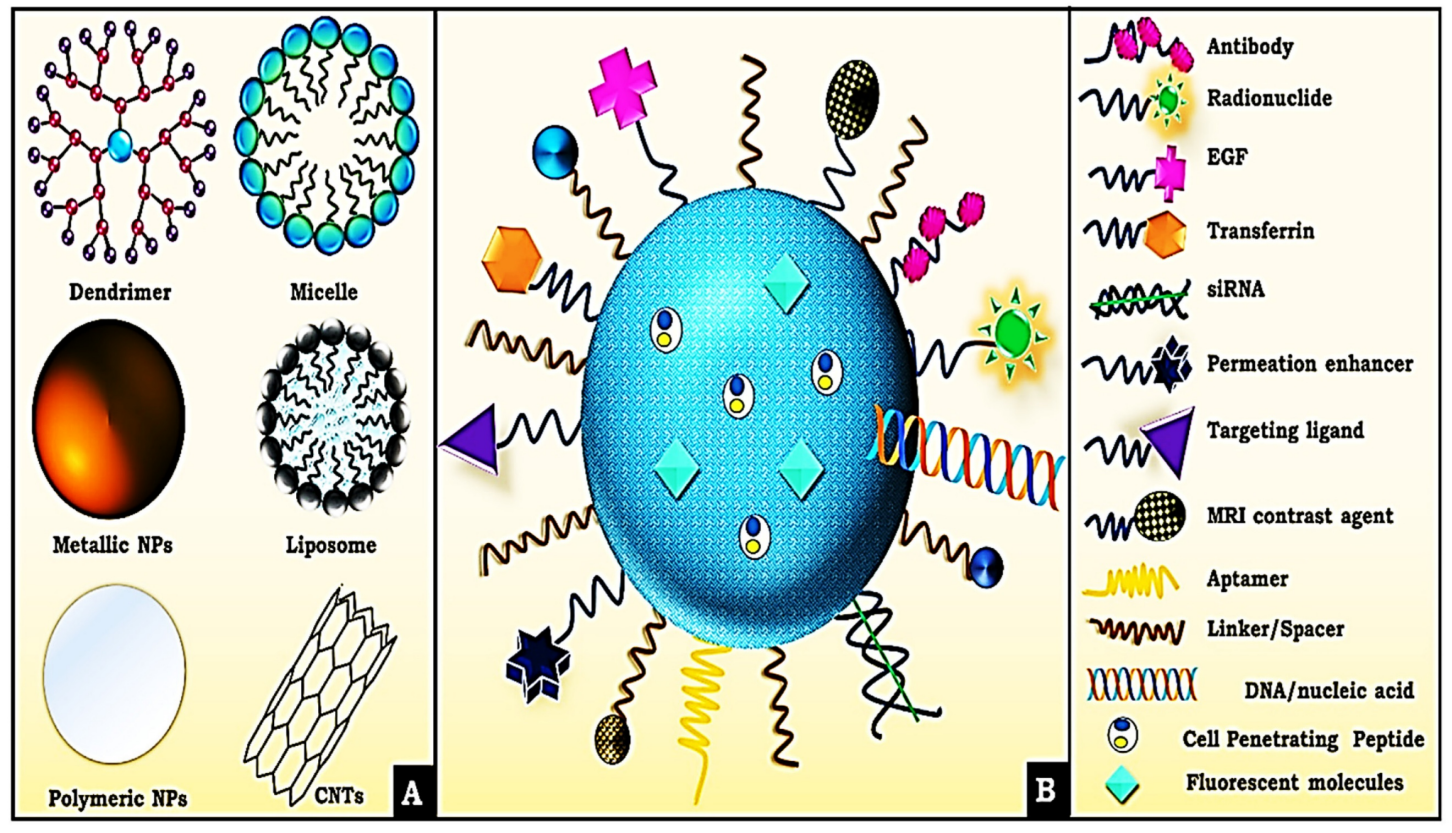
A- Delivery Vehicle used by siRNA. B- Various surface modifications used upon nano-conjugates for efficient delivery of siRNA. Adapted with CC by 4.0 permission from 66.

The capacity of siRNA to penetrate the lipophilic cell membrane is greatly reduced because of its large size and negatively charged backbone. For this reason, a proper delivery vehicle is necessary. The various modes of delivery for siRNA are illustrated below (Figure 4). Under *in vivo* conditions, the delivery vehicle protects the siRNA against various threats such as enzymatic degradation and phagocytosis. The delivery vehicle can target certain cells or increase the duration of circulation within the body via the inclusion of various surface moieties. Despite the effectiveness of the delivery vehicles in various aspects mentioned above, the material used for delivery can significantly activate the immune system 59-61. The delivery vehicles help the siRNA cross the membrane and pass through various subcellular compartments either via systemic or local administration (Figure 4). This eventually leads to the cytoplasm via various mechanisms during which the RNA gains exposure to several PPRs depending on the mechanism used.

**Cationic NMs:** Cationic NMs are a common choice for delivery vectors since they rapidly condense RNA due to electrostatic attraction 62. This delivery mechanism exposes the siRNA to various TLRs present in the endosomal compartments of various immune cells and PKR and RIG-I that are found in the cytoplasm. Therefore, this method of delivery triggers a higher immune response as compared to a delivery vehicle that doesn't traffic the siRNA via lysosomal and endosomal components 63.

**Lipid-based NMs:** Lipid-based NMs is another pivotal delivery system utilized for the delivery of siRNAs 64. siRNAs bind to the positive charge of lipid-based NMs via electrostatic attraction leading to the formation of lipo-complexes. C12-200 is a lipid-like delivery medium that achieves cellular entry *in vitro* via pinocytosis. This may reduce immunostimulatory activity induced by TLR since pinocytosis bypasses the lysosomal or endosomal pathway 65. The severity of the immune response and subsequent cytokine induction greatly depends on the charge, size, and *in vivo* biodistribution of the nanoparticle used for delivery. In a similar study, delivery of siRNA into the blood cells of humans was found to trigger IFN-α when a stable nucleic acid-lipid particle (SNALP) encapsulated polyethyleneimine-complexed siRNA was used however, usage of polysine, which led to formation of larger delivery particles led to induction of IL-6 and TNF-α 24.

**PGLA NMs:** PGLA-based NMs are one such delivery tool that is used due to their high retention capacity for siRNA and enhanced bioavailability 66, 67. PGLA-based nanoparticles, modified with tumor antigens, like ovalbumin (OVA/SOCS1 siRNA), were targeted using siRNA for SOCS1 gene in *in vitro* study, which led to tumor regression on bone marrow-derived dendritic cells via upregulation of immunostimulatory cytokines like TNF-α, IL-6, IL-12, and IL-2 68.

## 3. Influence on Host-Immune Response via siRNA

**Figure 5:**
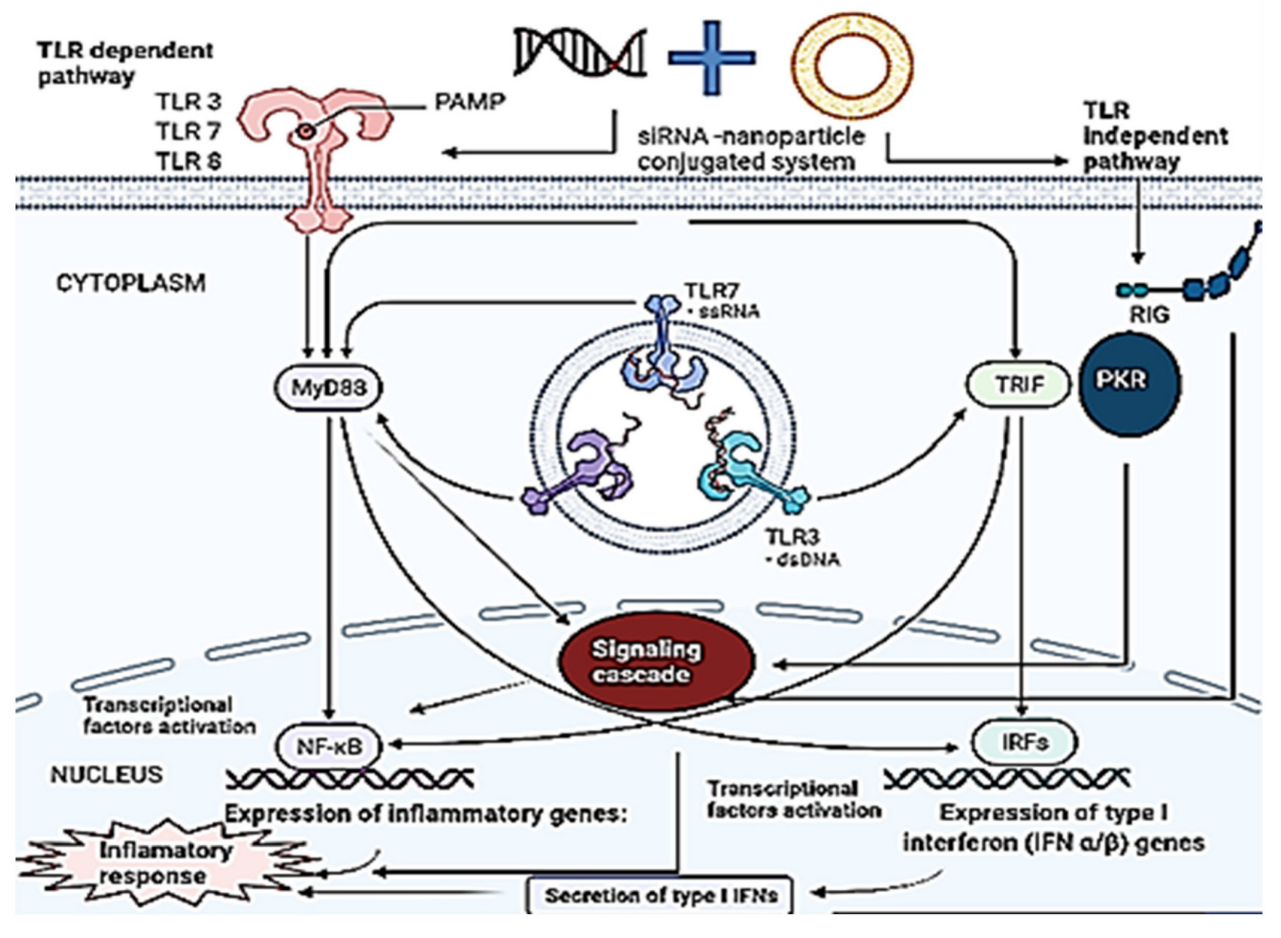
Immune activation of TLR-dependent and independent pathways by nano-based combinational therapy. Multiple PRRs are stimulated by siRNA upon delivery by nanoparticles. TLR3, TLR7, and TLR8 causes sequence-dependent immune activation by siRNA receptors in a MyD88-dependent manner. On the other hand, TLR-independent activation leads to immune activation through RIG, devoid of the sequence for siRNA. When each receptor is activated, it triggers a distinct immunological signaling cascade that enhances mRNA transcription, leading to an inflammatory response. The activation of NF-κB induces the production of inflammatory cytokines, and similarly, the activation of IRFs induces the production of type 1 interferon (IFNs).

There is a complex network of the immune system, protective cells that have developed over time. The following sections highlight the therapeutic features of the immune system upon activation by siRNA by activating innate and adaptive immunity. The innate immune responses of TLR and non-TLR pathways are elucidated below (Figure 5).

### 3.1 Effect of siRNA on the immune system

When naked exogenous RNA is delivered into the body, they are detected by the innate immune system, which is sub-divided into anti-viral response and acute inflammatory response. The inflammatory response leads to the induction of cytokines, including IL-6, IL-1, IL-12, and TNF-α, that serve as a link between adaptive and innate systems since they trigger the development of B cells, T-cells, and NK cells 69. TNF-α triggers inflammatory responses by inducing apoptosis, thus preventing viral multiplication 70. The pro-inflammatory cytokine milieu promotes phagocytosis by which foreign pathogens are killed and ingested 71. The antiviral arm of the innate immune system is characterized by the release of IFN type 1 via IFN-α and IFN-β. Along with this, there is an upregulation of over 100 anti-viral genes resulting in an antiviral state 72. The antiviral genes thus released are capable of producing NK cells and memory T cells. Thus, both the inflammatory and anti-viral arms of the innate immune system can kill infections and trigger an adaptive immune response 73. The recognition of PRRs, which were not present inside the host cells, triggers an innate immune response. These pattern recognition sequences can identify molecules that are often found in pathogens known as pathogen-associated molecular patterns, also called PAMP, or the molecules that an injured cell releases, known as damage-associated molecular patterns or DAMPs 74. Accordingly, over time, the innate immune system has evolved to incorporate numerous PRRs that identify various features of RNA structure causing immune stimulation on delivery of the siRNA, which is very difficult to avoid 75.

### 3.2 Effect of siRNA on TLR and non-TLR dependent pathways

Toll-like receptors (TLRs) belong to a class of PPR and could be identified as structurally conserved portions of foreign pathogens. There are ten functional TLRs in humans out of which TLR 7 and TLR8 recognize ssRNA whereas only TLR3 recognizes dsRNA 75. A horseshoe-like structure known as TLR-3 assists in recognizing dsRNA, which is a common feature of viral replication observed in apoptotic or lysed cells infected by the virus 37, 76, 77. In humans, TLR3 is historically found in both the cell surface of fibroblast and epithelial cells and the endosome of mature dendritic cells 76, 78. The expression of TLR3 is variable across cell lines and species and the immunostimulatory cascade triggered by TLR3 culminates in enhanced IFN-α and IFN-β production 79.

TLR7 is a key PRR solely located in the endoplasmic reticulum of B cell and plasmacytoid dendritic cells along with intercellular vesicles such as lysosome and endosome for identification of ssRNA in a sequence-specific manner 76, 80. siRNA is made up of two strands of ssRNA which elicits sequences depending upon TLR7 response 24, 43, 48. Activation of TLR7 in endosomes triggers a signal cascade which results in upregulation of IFN-α and IFN-β whereas, activation of TLR7 in lysosomes leads to the induction of IL-12 and TNF-α 61, 81. Activation of TLR7 in B cells triggers the adaptive immune system causing B cells to differentiate into plasma cells 80.

TLR8 detects ssRNA in a sequence-dependent manner like TLR7 by responding to both GU-rich and AU-rich motifs 32, 82. It is only expressed within intracellular vesicles and found solely in cells of myeloid lineage such as mDC, macrophage, and monocytes. Upregulation of TLR8 leads to the activation of the same molecules as TLR7 albeit there is a difference in the relative expression 83. Production of IL-6 and IL-1 during TL8 activation enhances the immune response 84.

The immune response against siRNA can be mediated by other proteins in the cytoplasm apart from TLR, which mainly protects endosomal compartments from infections caused by pathogens. PKR, is present in the cytoplasm of a large number of mammalian cells and can respond to as small as 11 bp of dsRNA in a sequence-independent manner 85-87. The exact characteristics of siRNA that PKR detects are not yet fully known, yet both conventional and blunt siRNA have been found to activate PKR to a considerable extent 86, 88, 89. Activation of PKR inhibits translation and triggers interferon response 85, 90.

Retinoic acid-inducible gene 1 protein (RIG-I) is an RNA helicase protein that identifies and acts in a non-sequence-specific manner against RNA 91, 92. RGI is found in mDCs and fibroblasts, which activates an immunological cascade inducing a high interferon response. The uncapped 5-triphosphate present in ssRNA or dsRNA present in most viral sequences is identified by RGI 21. The blunt-ended siRNA is independent of the sequence that can trigger RGI for immune response. The synthetic siRNA inserted *in vitro* frequently develops blunt ends and thus can be detected by RGI.

Thus, the immune system in humans is conditioned to perceive siRNA as a foreign entity, causing various PRRs present at different cellular sites to trigger an anti-inflammatory response in both TLR-dependent and independent ways. siRNA present in the cell surface is recognized by TLR3, sub-cellular compartments by TLR 3, TLR7, and TLR8, and those in the cytoplasm by PKR and RIG-I. The expressions of PPRs also seem to fluctuate over time as the environmental conditions change, and thus, the potential of siRNA to stimulate immune response should be considered while designing novel methods for siRNA delivery.

## 4. Intracellular Factor Modulation of siRNA-Nano-Conjugates Efficiency

**Figure 6:**
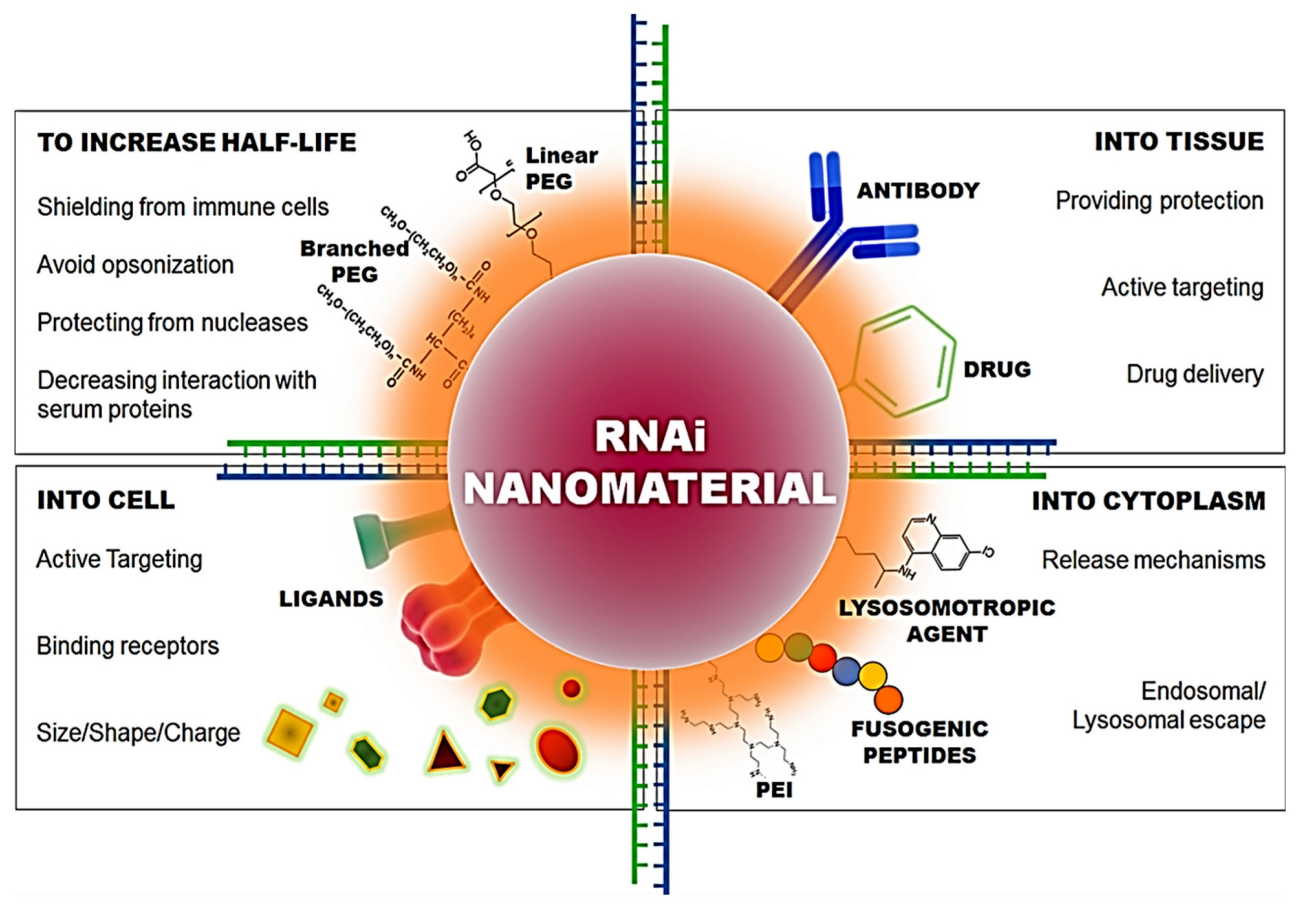
Characteristics required in nano-conjugates for the delivery of siRNA into the cell and cytoplasm. Adapted with permission from 93.

Combinational therapy of nano-conjugate-delivered siRNAs plays a critical role in immunomodulation either by enhancing the immune response or by impeding the suppression of the immune response. Designing an appropriate combinational therapy is essential for obtaining an immune response as the following factors determine the efficacy of siRNA in cancer immunotherapy (Figure 6).

### 4.1 Size and material of siRNA-loaded cargoes

The size of the nanoparticles is crucial to ensure proper delivery into tissues and is usually in the range of 10-100nm 94. The size range is based on *in vivo* clearance, biodiversity, and toxicity. Naked exogenous siRNA or nanoparticles with a size less than 10nm are subjected to excretion due to renal clearance from the blood compartment 95. To retain the nanoparticles longer in the blood, they are often modified chemically. On the other hand, particles larger than 15µm are eliminated by the reticuloendothelial system (RES) in the liver and spleen. Therefore, the delivery of the nanoparticles to the target is crucial as well as challenging 96. The uptake of nanoparticles is usually carried out by the macrophages, which in turn depends on factors like size, charge, etc. To enhance the retention time of nanoparticles in circulation, chemical modification can be carried out. Naked siRNA was subjected to renal clearance, with a half-life of less than 5 minutes in blood, but in the case when conjugated with cholesterol, the half-life of the siRNA could increase to a minimum of 30 minutes, due to the enhanced chemical stability 97. It was also observed that rapid removal of siRNA from the circulation takes place when conjugated with a cationic polymer. The Glomerular basement membrane (GBM) could break down cationic cyclodextrin-containing polymer (CDP)-based siRNA nanoparticles, allowing them to be excreted from circulation swiftly 98. Hence, engineering appropriate nanoparticles is crucial to ensure their uptake by the target cells.

### 4.2 siRNA administration

**Figure 7:**
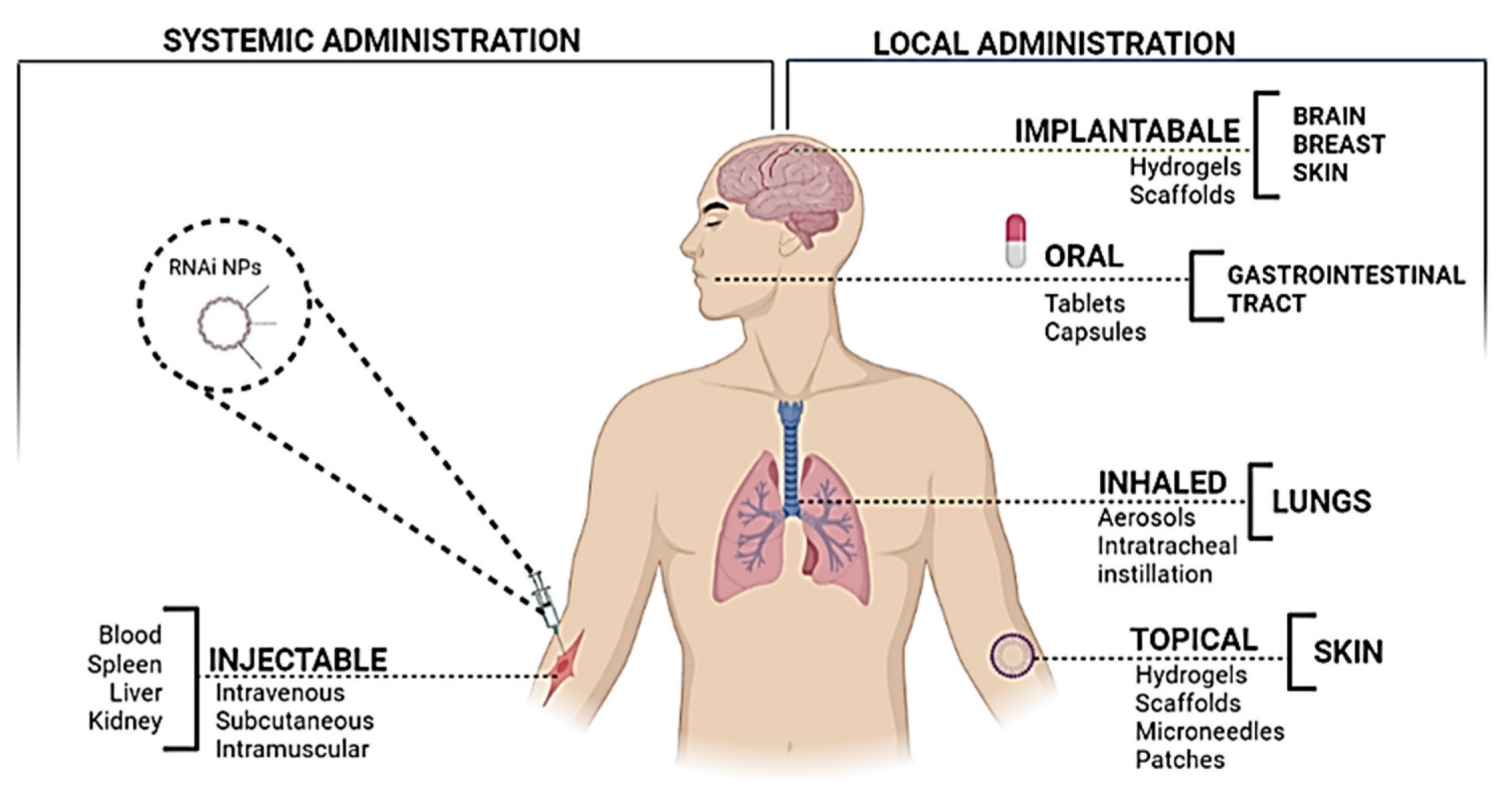
RNAi nanoparticles' administration routes and possible target organs to reach the target tissue and so interact with the local immune or tumor cells.

The most common and convenient route used for the administration of therapeutic agents is systemic delivery as it can not only reach the site of interest but it is non-invasive as well (Figure 7). However, in the case when the target organ is not the kidney or liver, it poses a challenge due to the lack of specificity 99. The siRNA, when administered systematically, is capable of inducing a non-specific immune response through the TLR-7 pathway or TLR-3 43. For *in vivo* delivery of siRNA, it was noticed that this effect could be reversed by chemical modifications, by incorporating 2′-O-methyl modifications into the sugar structure of specific bases in sense as well as anti-sense strands 100*.* Several hurdles are encountered during cancer therapy, which include the ability of the cancer cells to create an immune-protective or TME and the induction of various mechanisms for immunosuppression 101. Hence, even though the immunostimulatory effect of gene silencing using siRNA may raise certain questions about its efficacy due to its non-specificity, it seems to play a role in overcoming the limitation of immunosuppression. Therefore, to curb all the limitations, efficient designing of the nanoparticles is extremely critical.

### 4.3 siRNA delivery and endosomal escape

**Figure 8:**
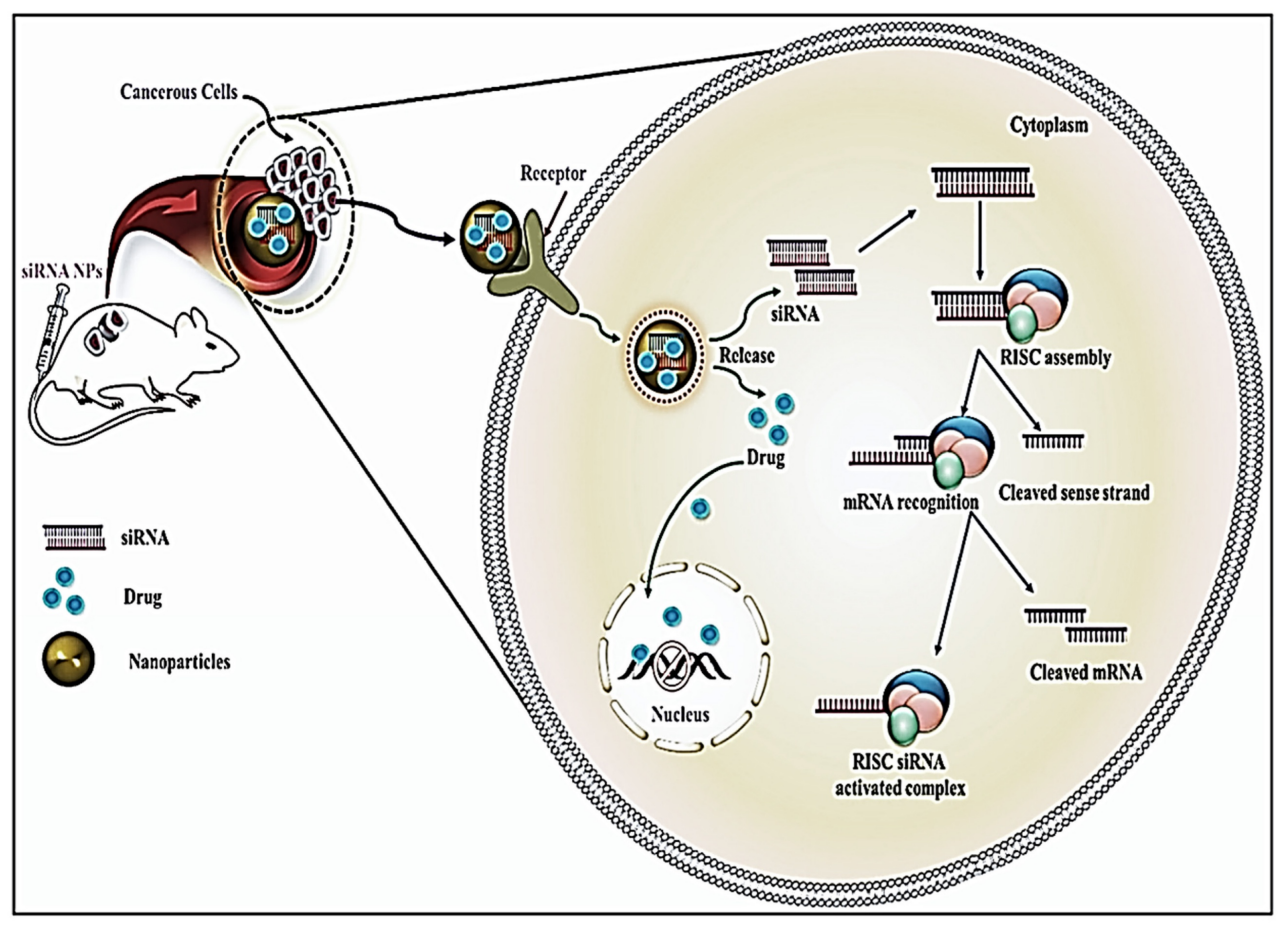
Mechanistic view of the delivery of siRNA by Nano-conjugates in cancer cells. Adapted with CC by 4.0 permission from 67.

The delivery of the siRNA to the cytoplasm is essential, but it poses certain challenges as well. The size and the charge of the nanoparticles are extremely crucial to enable the siRNA to cross the cell membrane 102. The processes of endocytosis and exocytosis of the nanoparticles depend on the shape, size, and charge 103. In the case of positively charged smaller nanoparticles, the uptake is carried by clathrin-dependent endocytosis, owing to the adsorption of the positively charged nanoparticle with the negatively charged membrane 103 (Figure 8). On the other hand, in the case of larger nanoparticles, the uptake is mediated by receptor-independent endocytosis 104. Along with the size and charge of the nanoparticles, the route of uptake plays a crucial role in the process of delivery as well 104 (Figure 8). The uptake of the smaller particles is facilitated through pinocytosis in dendritic cells which poses a high ability for antigen presentation. On the other hand, the larger particles are prone to phagocytosis by the macrophages, which have a low ability for antigen presentation 104.

A nanocarrier system based on human monoclonal prostate-specific membrane antigen-antibody (PSMAab) for targeted delivery of tripartite motif-containing 24 (TRIM24)-siRNA has been used not only to protect siRNA from enzymatic digestion but also for efficiently delivering siRNA in preclinical *in-vitro* model. The knockdown of TRIM24 by TRIM24-siRNA suppressed the proliferation *in vitro* and inhibited tumor growth of xenografts and bone metastasis model *in-vivo* as well 105.

In a similar study, the uptake of gold nanoparticles by macrophages can occur by various routes through pinocytosis or phagocytosis so that the cells could access another route if one happened to be blocked 106*.* In case of immunotherapies targeted against TAMs conducted on lung cancer in an *in vivo* murine model by administering it via intratracheal instillation, the administration of anti-VEGF siRNA gold nanoparticle for lung cancer could not only result in a dramatic reduction of TAMs in the tumor but decrease the size of the tumor as well. The dose of siRNA required is typically low, and the survival of the mice with lung tumors is increased. When applied with targeting M2 peptide, long-term removal of the tumor was observed 107. Thus, the delivery route of siRNA is essential.

The next challenge encountered in the process of the delivery of nanoparticles into the cytoplasm is the release of the nanoparticles from the endosome 108. In certain cases, the nanoparticles may get trapped within the endosome, which may later fuse with the lysosome, which would destroy the siRNA. These complexes must escape through the endosomal membrane to reach the cytoplasm, where all of the RNAi machinery is located, to silence genes 109. The fusion of viral envelopes with host cell endosomal membranes, which happens during viral infections, is one of the mechanisms devised to promote endosomal escape. The fusion domain of the influenza virus has been used to create several synthetic fusogenic peptides 110. Moreover, Stearyl atedoctaarginine lipid-based nanoparticles, modified with pH-dependent fusogenic peptide (GALA) were also observed to target SOCS1 using *in vitro* and *in vivo*, leading to STAT-1 phosphorylation, and increased the expression of the immunostimulatory cytokines due to the knockdown of SOCS1gene by siRNA for anti-cancer therapy. Endosomal escape was also enabled in this case since pH-dependent fusogenic peptide (GALA) had been altered on the lipid mixture, which in turn had been optimized to ensure endosomal fusion 111. Hence, the designing of the nanoparticles should rely on the type of immune cells that are intended to be targeted.

### 4.4 Circulation time and stability of siRNA

To protect the siRNA from serum nuclease and low chemical stability, alterations in the nanoparticles have been implemented by entrapping the siRNA within the nanoparticle 112. Enhanced circulation time and chemical stability can be achieved by attaching the siRNA to the surface of the nanomaterial, which renders the siRNA less vulnerable to degradation from the activity of the nucleases, thus ensuring higher uptake by cells 113. However, it might not be sufficient to shield the siRNA from clearance, as serum opsonin proteins may get adsorbed to the nanoparticle surface, and further mark it for the uptake by mononuclear phagocyte system (MPS), preventing it from reaching its target 18*.* To protect the surfaces from clearance, nanoparticles are conjugated with hydrophilic polymers. PEG, acrylic acid, maleic anhydride, and acrylamide polymers and copolymers, as well as allylamine and ethyleneimine, are the most commonly used hydrophilic polymer, which increases the circulation time of the nanoparticles by avoiding being bound to the serum proteins and hence escaping clearance. The CD206 gene, which is a mannose receptor and gets upregulated in TAMs, was targeted using mannosylated polymeric micelles in an *in vitro* study. It facilitated the delivery of the siRNA to the primary macrophages for its knockdown. This enhanced the delivery to up to 13-fold compared to that of free siRNA for anti-cancer therapy 114.

### 4.5 Targeting cell internalization

The siRNA must reach the target cells after reaching the target tissue, leaving the healthy cells unaffected. This could potentially be achieved with the help of specific ligands that can mediate internalization by the target cells, and the markers expressed specifically on the cancer cells can act as such a ligand 115. Moreover, the charge of the nanoparticle plays a key role, as well as the uptake of the nanoparticles with a positive charge on the surface, which was higher by the cancer cells, dendritic cells, and macrophages than that of others 115. This is due to the presence of a negative charge on the surface of the cell membrane, enabling an electrostatic interaction to take place. However, the non-specific uptake increases as well, along with the decrease in the half-life in circulation 115. In contrast, nanoparticles that carry a negative charge on the surface have a decreased rate of internalization. In this case, however, the half-life is much longer, enabling the nanoparticles to circulate longer. As a result, these nanoparticles can accumulate better at the sites of the tumor cells 116.

## 5. Host Immune System Activation through Combinational Immunotherapy of siRNA and Nano-Conjugates

**Figure 9:**
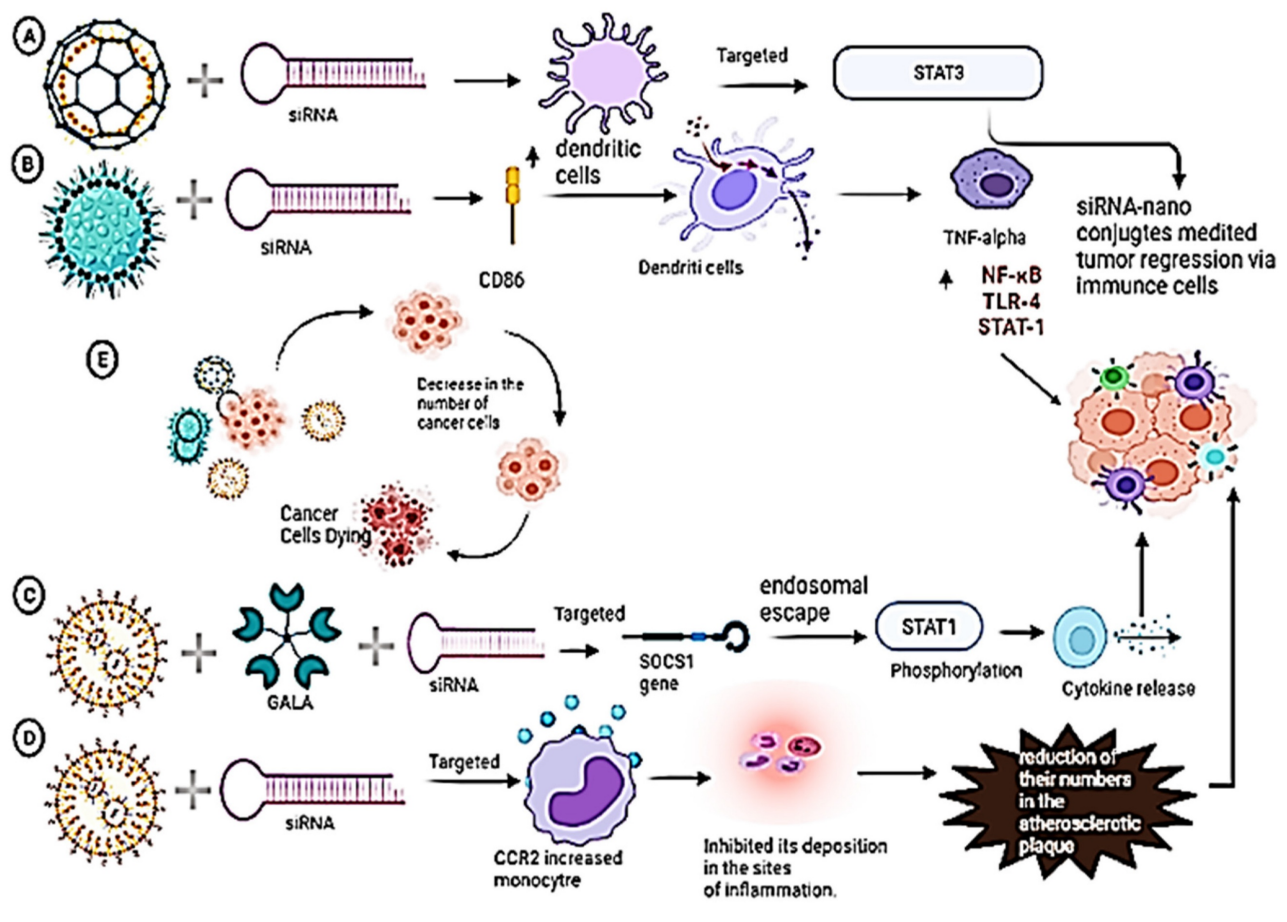
Immuno-stimulation of immune cells (dendritic and monocyte) via siRNA upon delivery by nano-conjugates. A) PLGA nanoparticles are employed to deliver siRNA into dendritic cells because of the ability of siRNA to knock down the expression of the desired gene STAT3, causing tumor regression via immune-stimulation of immune cells. B) PIE nanoparticles are employed to deliver siRNA, which upregulates the CD86 protein on the dendritic cells. This, in turn, will affect the TNF alpha cytokine to upregulate NF-KB, TLR-4, and STAT1 for tumor regression via immune stimulation. C) Lipid-based nanoparticles are employed with GALA peptide as a ligand to deliver siRNA. The target protein is SOCS1, which gets silenced by siRNA. This, in turn, causes the endosomal escape of SOCS1, which phosphorylates STAT1 Gene to release cytokines which in turn mediates tumor regression. D) Lipid-based nanoparticles are employed to deliver siRNA into monocytes which inhibits its deposition in the sites of inflammation and which leads to the reduction of their numbers in the atherosclerotic plaque which in turn mediates to tumor regression.

In the tumor microenvironment (TME), myeloid cells, including dendritic cells, TAMs, and MDSCs, play a pivotal role in immunosuppression, leading to tumor progression 117. These mentioned sub-sets of the population of immune cells can be targeted by nanoparticles, which may downregulate the immunosuppressive cytokines and transcriptional factors and kill cancer cells 118. This could, in turn, shift the tumor microenvironment to an anti-tumoral one. In the studies conducted with RNAi nanoparticles for immunotherapy, DCs are targeted in most cases. A summary of preclinical studies of immune cell stimulation via siRNA upon delivery by nanocarriers for tumor regression is elucidated in Table 1 and graphically portrayed below (Figure 9).

**Lipid-conjugated siRNAs:** Lipid-based nanoparticles have also been used in immunotherapies targeting monocytes owing to their enhanced stability 66. In an *in vivo* study using lymphoma-grafted mice administered systemically, the CCR2 (which is a chemokine receptor) gene was targeted by siRNA, which inhibited its deposition in the sites of inflammation, by degrading the CCR2 mRNA in monocytes. This, resulted in the reduction of their numbers in the atherosclerotic plaque, therefore lowering the volume of tumors, as well as the monocyte numbers 119.

Lipid-based nanoparticles could be used to target the genes for PD-L1 and PD-L2, which is effective in the knockdown of the PD-L gene using siRNA on human monocyte-derived dendritic cells. The phenotype of the dendritic cells was not altered, and the CD8+ response was observed to be enhanced for anti-cancer therapy in *ex vivo* transplant cancer patients 120. Similarly, an *in vitro* study was conducted with lipid-envelope type nanomaterial (MEND), which has been modified with R8 and pH-dependent fusogenic peptide (GALA), targeting the gene A20 in a siRNA-dependent manner. There is simultaneous stimulation of LPS that takes place along with the enhancement of A-20 silenced in dendritic cells, leading to the production of enhanced immunostimulatory molecules for anti-cancer therapy 121.

**PEI-conjugated siRNAs:** Numerous studies were conducted using PEI-based nanoparticles to target the dendritic cells owing to their bio-compatibility and non-immunogenic nature 66. Silencing of PD-L1 resulted in the shifting of the tumor-associated regulatory dendritic cells to the normal dendritic cells, thus enhancing the immunostimulatory cytokines when a PEI-based nanoparticle was injected peritoneally in *in vitro*, as well as *in vivo* ovarian cancer murine model 59. In another study, STAT-3 was silenced using siRNA in *in vitro* and i*n vivo* lymphoma mouse models by stearic acid-modified nanoparticles (PEI-StA), leading to the restoration of the usual properties of the dendritic cells, which was capable of promoting cytotoxic T lymphocyte (CTL) activity 122.

## 6. Impact of Combinational Therapy (siRNA-nanoparticles) for Enhanced Anti-Cancer Therapy

**Figure 10:**
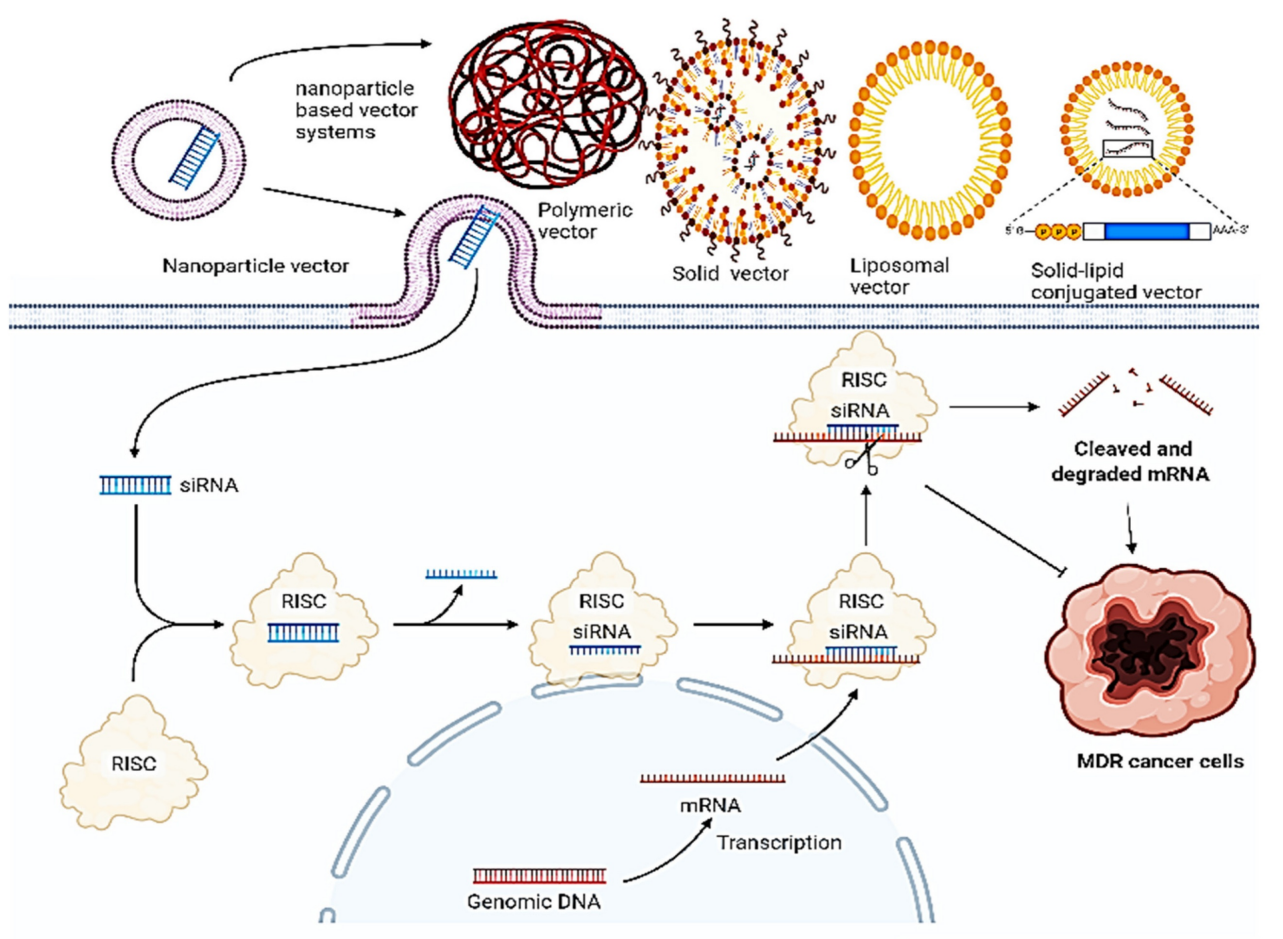
Systemic delivery of siRNA via different nanoparticle-based units for reduced tumor regression. Various nanoparticles such as solid NPs, liposomal NPs, lipid solid NPs, and polymeric NPs are used to deliver siRNA with each one having a different loading mechanism. siRNA-mediated silencing or knowing down of the MDR gene works on the above-discussed principle.

Drug resistance in cancer remains an issue concerning the failure of chemotherapeutics, leading to tumor recurrence and metastasis. A combination of these therapies, particularly by targeting genes that are involved in drug resistance in a siRNA-dependent manner post-delivery upon nanoparticles, has emerged as a novel strategy for cancer therapy. The mechanism upon which this synergistic action is based is elucidated below (Figure 10), and their pre-clinical significance and relevance is synopsized in Table 2.

### 6.1 PEG-based nano-conjugates for siRNA delivery

PEG-based nano-conjugates offer a promising enhanced delivery of siRNAs with biocompatibility and ease of production. As a result, in recent times, PEG base nano-conjugates have been employed in several studies to deliver siRNAs 66. PEG-PAMAM/VEGF siRNA dendriplexes displayed efficient gene silencing and inhibited vascular-like formation (angiogenesis) in retinal vascular endothelial cells for reduced tumor volume 125. An arginine-grafted, bio-reducible poly(cystaminebisacrylamidediaminohexane), called ABP, and PAMAM (PAM-ABP) were used for delivering anti-VEGF siRNA to various cancers for tumor regression 126.* TWIST* is a transcription factor involved in drug resistance of ovarian cancer and the combination of PAMAM dendrimers along with mesoporous silica nanoparticles were used to carry the siRNA targeting *TWIST* gene for down-regulation of *TWIST* mRNA to overcome cisplatin-resistant ovarian cancer cells by increasing the sensitivity of cancer cells for chemotherapeutics to cause tumor regression 127, 128. Similarly, co-administration of the nanoparticles with siRNA and paclitaxel caused a reduced tumor size and mortality via intra-tumoral injection in tumor-bearing mice 129.

In a similar study, a dendrimer based on enzymatically synthesized glycogen (ESG) with a Quaternary ammonium group was introduced via an epoxide ring-opening reaction alongside glycidyl tri-methyl-ammonium chloride (GTMA). These polymers were bound by siRNA targeted for superoxide dismutase 2 (Sod2) via electrostatic interactions, causing downregulation of this mitochondrial antioxidant enzyme and increased susceptibility to chemotherapeutically induced redox damage, resulting in ovarian clear cell carcinomas based on tumor regression 130.

A biodegradable polymeric matrix has been used as a vehicle for the delivery of siRNA with a drug. The drug, siG12D LODER, was used, which is a miniature biodegradable polymeric matrix containing an anti-KRASG12D siRNA (siG12D) drug designed to ensure the release of the drug regionally within the pancreatic tumor at a prolonged rate. Since most of the cases of Pancreatic ductal adenocarcinoma contain KRAS oncogene, this drug is expected to result in silencing and hence in the inhibition of cancer growth (NCT01188785).

Thus, based on the above-listed studies it would be suggestive that PEG-based nano-conjugates cause enhanced anti-cancer therapy due to the targeted delivery caused by the nano-conjugates. Even though further studies need to be conducted in this direction, it appears as a promising combinational theragnostic therapy.

### 6.2 Lipid-based nano-conjugates for siRNA delivery

Lipid-based nano-conjugates have gained tremendous attention in recent times due to their high stability and ability to deliver cargo in a pH-responsive manner 66. A delivery system composed of Glu-urea-Lys PSMA-targeting ligand/siRNA was developed to be incorporated into a lipid nanoparticle for targeting androgen receptors and inhibiting tumor cellular proliferation on the surface of PCa cells 131. Similarly, combinations of LHRHPEG- siRNA and an anti-cancer drug-MSN were delivered via liposomal nanoparticle complex for anti-cancer therapy. The mixture of LHRHPEG-siRNA (*BCL2*)-MSN and LHRH-PEG-siRNA (*MRP1*)-MSN provided effective downregulation of *BCL2* and *MRP1* mRNA levels *in vitro,* leading to tumor regression 132.

A liposomal complex was prepared by mixing siRNA conjugated with DOTAP/cholesterol, which contains Apolipoprotein A or recombinant human ApoA-1 for anti-cancer therapy 133. DOTAP-cholesterol was intravenously delivered by TUSC2 in phase 1 of the clinical trial 134. The tumor suppressor gene showed gene expression and alterations in TUSC2-regulated pathways *in vitro* and *in vivo* studies for reduced tumor volume in lung cancer (NCT00059605). Folic acid molecules (FNP) were used to modify the liposomal complex along with DOTAP-chol for targeting siRNA delivery in folate receptor-overexpressing lung cancer cells for tumor regression 135. Amino-PEG is mostly conjugated by folate and is incorporated into the bilayer of the liposomes for targeting VEGF via siRNA delivered by synthesizing bio-reducible PEI (SS-PEI) polymer for treating liver cancer *in vivo* murine model for tumor regression. This PEI-based siRNA nanoparticle system downregulated VEGF and inhibited liver tumor growth 136.

DCR-MYC was used as a novel synthetic dsRNA in a stable lipid particle suspension, which is responsible for targeting the oncogene MYC. Since the activation of MYC is required for tumor growth, this study proposes to inhibit advanced cancer growth (NCT02110563). Similarly, in another trial, DCR-MYC was used to inhibit hepatocellular cancer growth. It was administered to patients by intravenous infusion, and this study had undergone Phase 2 trials with reduced tumor volume and increased survival (NCT02314052).

Atu 027 is a liposomal-siRNA formulation that was used to inhibit protein kinase in the vascular endothelium. It had been administered as a single treatment, which was followed by its use as repeated treatment and was applied as therapy to numerous patients with advanced solid cancer, and lipid nanoparticles had been used as vehicles for delivery. This study had undergone the completion of the Phase 1 trials (NCT00938574).

Thus, based on the aforementioned pre-clinical and clinical studies it would be suggested that delivery of siRNA alone or in combination with anti-cancer drugs via lipid nano-conjugates provides a promising avenue for developing more therapeutic ventures in this direction.

### 6.3 Metal-based nano-conjugates for siRNA delivery

Metal-based nano-conjugates have gained a lot of attention in recent times due to their ability to deliver targeted siRNA. In a similar study, Ruthenium-based nanoparticles were used to deliver siRNAs in combination with a metal-organic framework to exhibit significant downregulation of P-gp and VEGF as compared to single siRNA, which suggests the enhanced gene silencing effects 137.

Similarly, using the combinational approach in ovarian cancer, the *NOTCH3 gene* was knocked down, which encoded a marker involved in ovarian cancer recurrence and chemotherapy resistance. An aptamer-siRNA chimera was delivered using gold nanoparticles conjugated with iron (II, III) oxide and PEI to target the overexpressed protein known as vascular endothelial growth factor (VEGF). The aptamer successfully targeted VEGF signaling in cisplatin-resistant cells via a nanoparticle chimera delivery system, which effectively knocked down the NOTCH3 gene for tumor regression in ovarian cancer 138. Moreover, Au-Fe_3_O_4_ heterogeneous nanoparticles were developed to deliver VEGF aptamer-Notch3 siRNA chimera specifically to VEGF-positive ovarian cancer cells to silence the target Notch3 gene and enhance the antitumor effects 139. Therefore, these findings suggest that MOF nanoparticles are a promising vector for the delivery of siRNA and effective therapeutics for the treatment of drug-resistant cancers.

Thus, based on above mentioned pre-clinical study MOFs-conjugated siRNA offers an interesting avenue that would require further investigation to understand comprehensively but offer a novel avenue for siRNA-Nanoconjugates-based tumor regression.

## 7. Conclusion and Future Outlooks

Even though there has been a significant advancement in the understanding of cancer mechanisms, the clinical and preclinical trials done on patients and animal cell lines with the most aggressive tumors have scarcely improved in the last 40 years.

siRNA based therapeutics have widespread benefits in addressing several challenges posed to the chemotherapeutics in the present standard of care. Compared it to other therapeutic approaches, siRNA provides substantial benefits. It can accurately inhibit the expression of target genes, promote tumor cell apoptosis, and have fewer side effects than traditional treatment methods. The potential of siRNA-based drugs to remodel TME has also proven to be effective and could intervein with the core proteins in all components of TME. The use of siRNA in combination with nano-conjugates *in vivo* to silence specific genes will be a valuable tool for future approaches in cancer therapies. The combination of minimum delivery materials with higher therapeutic component loading can help prevent material-related toxicity and other undesirable consequences. However, the present pace of discovering new drug targets and enhanced engineering in nanoparticle design strengthen the areas of prospective development. However, in recent times, nano-CRISPR-based therapies are emerging as a novel avenue that may pose stiff competition to nano-conjugate-based siRNA delivery 146.

Some of the key challenges include: Nucleases are the primary degraders of SiRNA molecules in the blood stream restricting their target. Moreover, there is a scope for non-specific targeting which contribute towards issues in specificity. With regards to the immune system, SiRNA can induce inflammation and might affect therapy. Several other off-target effects are, issues with mRNA sequencing.

However, nanoconjugate-based siRNA therapy is expected to gain widespread acceptance and become a standard technique of cancer treatment soon. Several Phase I trials exploring the use of siRNA for the treatment of solid tumors have recently concluded. To present, nanoconjugate-based delivery methods have been used in all trials to deliver therapeutic siRNA to tumor tissue after systemic dosing. Despite worries about overstimulating the immune system after systemic siRNA treatment in people, the data on siRNA therapeutics so far has demonstrated that they are well tolerated, with very minor and treatable immunostimulatory effects.

## Figures and Tables

**Table 1:** Synopsis of the synergistic action of nanoparticle and siRNA for tumor regression via immune cell activation.

Nanoparticle type used in synergy with siRNA	Targeted immune Cell	*In-vivo*	*In-vitro*	Gene targeted by siRNA	Comments	Reference
PGLA-based	Dendritic Cells	Lymphoma mice		STAT3; Silencing	Ovalbumin-specific T-cell activity was observed when NP was administered to lymphoma mice. Growth of tumor restricted.	68
	Dendritic Cells		B16F10	STAT3; Silencing	CD86 expression upregulated, Increased release of TNF-α, Proliferation of allogenic T-cells	123
	Dendritic Cells		CD8 OVA 1.3 T cells	SOCS1	Increased levels of TNF-a, IL-6, IL-12, IL-2, More immunotherapeutic effects	68
PEI-based	Dendritic Cells	Lymphoma mice	B16 melanoma	STAT3; Silencing	Dendritic cells are enabled to perform CTL activity, Reduced growth of tumor in B16 mice.	122
	Dendritic Cells	Ovarian cancer mice	HEK293 cells	PD-L1; Silencing	Induction of TLR5 and TLR7, Restoration of the function of Dendritic cells in ovarian cancer mice.	59
	Dendritic Cells	Ovarian cancer mice		miR-155	Increased miR-155 activity, Anti-inflammatory mediators silenced, Restoration of CTL activity of Dendritic cells in ovarian cancer mice.	124
Lipid-based	Dendritic Cells		Hep3b cell line	PD-L1 PD-L2; Knockdown	Knockdown of PD-L expression	120
	Monocytes	Lymphoma graft		CCR2; Silencing	No deposition of CCR2 in sites of inflammation, Decrease in tumor volume	119
Lipid Envelope-based	Dendritic Cells		Cell line from Female C57BL/6 (H-2b) mice	A20; Silencing	LPS stimulation, Increase in immunostimulatory cytokines	121
Stearylatedoctaargininelipid-based	Dendritic Cells		HeLa cells and E.G.,7-OVA cells	SOCS1; Silencing	Increased STAT1 phosphorylation, Increase in immunostimulatory cytokines	111
Gold nanoparticles	Macrophages	Lung cancer mice	Mouse BALB/c macrophage J774.2 cell line	VEGF; Silencing	Decrease in TAMs in lung tumor tissues, Decrease in tumor size, Enhancement of survival in mice	107
Mannosylated Polymeric Micelles	Macrophages		Breast cancer cell line	CD206; Knockdown	Improved delivery of the siRNAs, 90% knockdown	114

**Table 2:** Preclinical and clinical studies involved in combinational therapy for inhibiting various stages of MDR cancers.

Type of Cancer	siRNA	Nanoparticle	*In Vitro*	*In Vivo*	Clinical Trails	Hallmark Modulation	Reference	
Lung Cancer	MRP1 + BCL2	Cationic lipids - DOTMA and N-[1-(2,3-dioleoyloxy) N,N,N-trimethylammonium methylsulfate (DOTAP)	H69AR cell line,MCF-7/ AD cell line, andHCT15 cell line.			Metastasis	140	


	MRP1 + BCL2	Pyridylthiolterminated MSN, as an inhalation delivery	None	A549 cell line, Tumor (A549)-bearingnude mice	Phase 1	Invasion	135	

Prostate Cancer	TRIM24 gene-specific siRNA	Nanocarrier system based on humanmonoclonal prostate-specific membrane antigen-antibody (PSMAab) for targeted delivery of tripartiteMotif-containing 24 (TRIM24)-siRNA.	PSMA + CRPC cells.			Invasion	105	


	GRP-78 specific siRNA;	Calcium phosphate core, dioleoyl phosphatidic acid, and arginine-glycine-aspartic acid peptide-modified polyethylene glycol, for co-delivery of the78-kDa glucose-regulated protein (GRP78)-specific siRNA and docetaxel as a combination therapy.	PC3-CRPC	PC3-CRPC		Metastasis	141	




	Si-TWIST (si419 and si494), a developmental transcription factor that leads to chemotherapic resistance and cancer cell stemness.	Amphiphilic PAMAM dendrimer YTZ3-15 or polyethyleneimine (PEI) coatedmesoporous silica nanoparticle (MSN)		A2780R and Ovcar8 cells; mice treated		Metastasis	128, 142, 143	
Breast Cancer	P-gp and VEGF-specific siRNA	Selenium/ruthenium-MOF nanoparticles		MCF-7/T cell; nude mice		Invasion and metastasis	144, 145	

## References

[1] Ortiz-Tudela E, Mteyrek A, Ballesta A, Innominato P, Levi F (2013). Cancer chronotherapeutics: experimental, theoretical, and clinical aspects. Circadian clocks.

[2] Sung H, Ferlay J, Siegel RL, Laversanne M, Soerjomataram I, Jemal A (2021). Global cancer statistics 2020: GLOBOCAN estimates of incidence and mortality worldwide for 36 cancers in 185 countries. CA: a cancer journal for clinicians.

[3] Tekade RK, Dutta T, Tyagi A, Bharti AC, Das BC, Jain NK (2008). Surface-engineered dendrimers for dual drug delivery: a receptor up-regulation and enhanced cancer targeting strategy. Journal of drug targeting.

[4] Liu B, Zhou H, Tan L, Siu KTH, Guan X-Y (2024). Exploring treatment options in cancer: tumor treatment strategies. Signal transduction and targeted therapy.

[5] Senapati S, Mahanta AK, Kumar S, Maiti P (2018). Controlled drug delivery vehicles for cancer treatment and their performance. Signal Transduct Target Ther.

[6] Liang X-J, Chen C, Zhao Y, Wang PC (2010). Circumventing tumor resistance to chemotherapy by nanotechnology. Multi-Drug Resistance in Cancer: Springer.

[7] Bock C, Lengauer T (2012). Managing drug resistance in cancer: lessons from HIV therapy. Nature Reviews Cancer.

[8] Bhattacharjee R, Mitra P, Gupta N, Sharma S, Singh VK, Mukerjee N (2022). Cellular landscaping of exosomal miRNAs in cancer metastasis: From chemoresistance to prognostic markers. Advances in Cancer Biology - Metastasis.

[9] Kesharwani SS, Kaur S, Tummala H, Sangamwar AT (2018). Overcoming multiple drug resistance in cancer using polymeric micelles. Expert opinion on drug delivery.

[10] Wilting RH, Dannenberg J-H (2012). Epigenetic mechanisms in tumorigenesis, tumor cell heterogeneity and drug resistance. Drug Resistance Updates.

[11] Shapira A, Livney YD, Broxterman HJ, Assaraf YG (2011). Nanomedicine for targeted cancer therapy: towards the overcoming of drug resistance. Drug resistance updates.

[12] Holohan C, Van Schaeybroeck S, Longley DB, Johnston PG (2013). Cancer drug resistance: an evolving paradigm. Nature reviews Cancer.

[13] Sousa C, Videira M (2025). Dual Approaches in Oncology: The Promise of siRNA and Chemotherapy Combinations in Cancer Therapies. Onco.

[14] Zhu Y, Zhu L, Wang X, Jin H (2022). RNA-based therapeutics: an overview and prospectus. Cell death & disease.

[15] Sinha A, Bhattacharjee R, Bhattacharya B, Nandi A, Shekhar R, Jana A (2023). The paradigm of miRNA and siRNA influence in Oral-biome. Biomedicine & Pharmacotherapy.

[16] Pai SI, Lin YY, Macaes B, Meneshian A, Hung CF, Wu TC (2006). Prospects of RNA interference therapy for cancer. Gene Therapy.

[17] Zhang J, Chen B, Gan C, Sun H, Zhang J, Feng L (2023). A comprehensive review of small interfering RNAs (siRNAs): mechanism, therapeutic targets, and delivery strategies for cancer therapy. International journal of nanomedicine.

[18] Kanasty R, Dorkin JR, Vegas A, Anderson D (2013). Delivery materials for siRNA therapeutics. Nature materials.

[19] Gao H, Cheng R, A Santos H (2021). Nanoparticle-mediated siRNA delivery systems for cancer therapy. VIEW.

[20] Kanasty RL, Whitehead KA, Vegas AJ, Anderson DG (2012). Action and reaction: the biological response to siRNA and its delivery vehicles. Molecular Therapy.

[21] Kato H, Takeuchi O, Sato S, Yoneyama M, Yamamoto M, Matsui K (2006). Differential roles of MDA5 and RIG-I helicases in the recognition of RNA viruses. Nature.

[22] Bridge AJ, Pebernard S, Ducraux A, Nicoulaz A-L, Iggo R (2003). Induction of an interferon response by RNAi vectors in mammalian cells. Nature genetics.

[23] Sledz CA, Holko M, De Veer MJ, Silverman RH, Williams BR (2003). Activation of the interferon system by short-interfering RNAs. Nature cell biology.

[24] Judge AD, Sood V, Shaw JR, Fang D, McClintock K, MacLachlan I (2005). Sequence-dependent stimulation of the mammalian innate immune response by synthetic siRNA. Nature biotechnology.

[25] Bhattacharjee R, Dey T, Kumar L, Kar S, Sarkar R, Ghorai M (2022). Cellular landscaping of cisplatin resistance in cervical cancer. Biomedicine & Pharmacotherapy.

[26] Kogan G, Šandula J, Korolenko TA, Falameeva OV, Poteryaeva ON, Zhanaeva SY (2002). Increased efficiency of Lewis lung carcinoma chemotherapy with a macrophage stimulator—yeast carboxymethyl glucan. International immunopharmacology.

[27] Uckun F, Ramakrishnan S, Houston L (1985). Increased efficiency in selective elimination of leukemia cells by a combination of a stable derivative of cyclophosphamide and a human B-cell-specific immunotoxin containing pokeweed antiviral protein. Cancer research.

[28] Ihde DC (1992). Chemotherapy of lung cancer. The New England journal of medicine.

[29] Myhr G, Moan J (2006). Synergistic and tumour selective effects of chemotherapy and ultrasound treatment. Cancer letters.

[30] Hornung V, Schlender J, Guenthner-Biller M, Rothenfusser S, Endres S, Conzelmann K-K (2004). Replication-Dependent Potent IFN-α Induction in Human Plasmacytoid Dendritic Cells by a Single-Stranded RNA Virus. The Journal of Immunology.

[31] Diebold SS, Massacrier C, Akira S, Paturel C, Morel Y, Reis e Sousa C (2006). Nucleic acid agonists for Toll-like receptor 7 are defined by the presence of uridine ribonucleotides. European journal of immunology.

[32] Heil F, Hemmi H, Hochrein H, Ampenberger F, Kirschning C, Akira S (2004). Species-specific recognition of single-stranded RNA via toll-like receptor 7 and 8. Science.

[33] Sioud M (2006). Single-stranded small interfering RNA are more immunostimulatory than their double-stranded counterparts: A central role for 2′-hydroxyl uridines in immune responses. European journal of immunology.

[34] Hornung V, Ellegast J, Kim S, Brzózka K, Jung A, Kato H (2006). 5'-Triphosphate RNA Is the Ligand for RIG-I. Science.

[35] Pichlmair A, Schulz O, Tan CP, Näslund TI, Liljeström P, Weber F (2006). RIG-I-Mediated Antiviral Responses to Single-Stranded RNA Bearing 5'-Phosphates. Science.

[36] Kim D-H, Longo M, Han Y, Lundberg P, Cantin E, Rossi JJ (2004). Interferon induction by siRNAs and ssRNAs synthesized by phage polymerase. Nature Biotechnology.

[37] Choe J, Kelker MS, Wilson IA (2005). Crystal structure of human toll-like receptor 3 (TLR3) ectodomain. Science.

[38] Elmén J, Thonberg H, Ljungberg K, Frieden M, Westergaard M, Xu Y (2005). Locked nucleic acid (LNA) mediated improvements in siRNA stability and functionality. Nucleic Acids Research.

[39] Dahlgren C, Wahlestedt C, Thonberg H (2006). No induction of anti-viral responses in human cell lines HeLa and MCF-7 when transfecting with siRNA or siLNA. Biochemical and Biophysical Research Communications.

[40] Fluiter K, Mook OR, Baas F (2009). The therapeutic potential of LNA-modified siRNAs: reduction of off-target effects by chemical modification of the siRNA sequence. Methods in molecular biology (Clifton, NJ).

[41] Judge AD, Bola G, Lee AC, MacLachlan I (2006). Design of noninflammatory synthetic siRNA mediating potent gene silencing in vivo. Molecular therapy: the journal of the American Society of Gene Therapy.

[42] Robbins M, Judge A, Liang L, McClintock K, Yaworski E, MacLachlan I (2007). 2′-O-methyl-modified RNAs Act as TLR7 Antagonists. Molecular Therapy.

[43] Hornung V, Guenthner-Biller M, Bourquin C, Ablasser A, Schlee M, Uematsu S (2005). Sequence-specific potent induction of IFN-α by short interfering RNA in plasmacytoid dendritic cells through TLR7. Nature medicine.

[44] Langkjaer N, Pasternak A, Wengel J (2009). UNA (unlocked nucleic acid): a flexible RNA mimic that allows engineering of nucleic acid duplex stability. Bioorganic & medicinal chemistry.

[45] Bramsen JB, Pakula MM, Hansen TB, Bus C, Langkjær N, Odadzic D (2010). A screen of chemical modifications identifies position-specific modification by UNA to most potently reduce siRNA off-target effects. Nucleic Acids Research.

[46] Kenski DM, Cooper AJ, Li JJ, Willingham AT, Haringsma HJ, Young TA (2009). Analysis of acyclic nucleoside modifications in siRNAs finds sensitivity at position 1 that is restored by 5′-terminal phosphorylation both in vitro and in vivo. Nucleic Acids Research.

[47] Morrissey DV, Lockridge JA, Shaw L, Blanchard K, Jensen K, Breen W (2005). Potent and persistent in vivo anti-HBV activity of chemically modified siRNAs. Nature Biotechnology.

[48] Sioud M (2005). Induction of inflammatory cytokines and interferon responses by double-stranded and single-stranded siRNAs is sequence-dependent and requires endosomal localization. Journal of molecular biology.

[49] Robbins M, Judge A, MacLachlan I (2009). siRNA and innate immunity. Oligonucleotides.

[50] Cekaite L, Furset G, Hovig E, Sioud M (2007). Gene Expression Analysis in Blood Cells in Response to Unmodified and 2′-Modified siRNAs Reveals TLR-dependent and Independent Effects. Journal of Molecular Biology.

[51] Deleavey GF, Watts JK, Alain T, Robert F, Kalota A, Aishwarya V (2010). Synergistic effects between analogs of DNA and RNA improve the potency of siRNA-mediated gene silencing. Nucleic Acids Research.

[52] Hamm S, Latz E, Hangel D, Müller T, Yu P, Golenbock D (2010). Alternating 2'-O-ribose methylation is a universal approach for generating non-stimulatory siRNA by acting as TLR7 antagonist. Immunobiology.

[53] Judge AD, Robbins M, Tavakoli I, Levi J, Hu L, Fronda A (2009). Confirming the RNAi-mediated mechanism of action of siRNA-based cancer therapeutics in mice. The Journal of clinical investigation.

[54] Furset G, Sioud M (2007). Design of bifunctional siRNAs: combining immunostimulation and gene-silencing in one single siRNA molecule. Biochem Biophys Res Commun.

[55] Jackson AL, Burchard J, Leake D, Reynolds A, Schelter J, Guo J (2006). Position-specific chemical modification of siRNAs reduces “off-target” transcript silencing. Rna.

[56] Leuschner PJF, Ameres SL, Kueng S, Martinez J (2006). Cleavage of the siRNA passenger strand during RISC assembly in human cells. EMBO reports.

[57] Chiu Y-L, Rana TM (2003). siRNA function in RNAi: a chemical modification analysis. Rna.

[58] Czauderna F, Fechtner M, Dames S, Aygün H, Klippel A, Pronk GJ (2003). Structural variations and stabilising modifications of synthetic siRNAs in mammalian cells. Nucleic Acids Research.

[59] Cubillos-Ruiz JR, Engle X, Scarlett UK, Martinez D, Barber A, Elgueta R (2009). Polyethylenimine-based siRNA nanocomplexes reprogram tumor-associated dendritic cells via TLR5 to elicit therapeutic antitumor immunity. The Journal of clinical investigation.

[60] Kornek M, Lukacs-Kornek V, Limmer A, Raskopf E, Becker U, Klöckner M (2008). 1,2-Dioleoyl-3-Trimethylammonium-Propane (DOTAP)-Formulated, Immune-Stimulatory Vascular Endothelial Growth Factor A Small Interfering RNA (siRNA) Increases Antitumoral Efficacy in Murine Orthotopic Hepatocellular Carcinoma with Liver Fibrosis. Molecular Medicine.

[61] Lee J, Chuang T-H, Redecke V, She L, Pitha PM, Carson DA (2003). Molecular basis for the immunostimulatory activity of guanine nucleoside analogs: activation of Toll-like receptor 7. Proceedings of the National Academy of Sciences.

[62] Cai X, Dou R, Guo C, Tang J, Li X, Chen J (2023). Cationic polymers as transfection reagents for nucleic acid delivery. Pharmaceutics.

[63] Akinc A, Thomas M, Klibanov AM, Langer R (2005). Exploring polyethylenimine-mediated DNA transfection and the proton sponge hypothesis. The Journal of Gene Medicine: A cross-disciplinary journal for research on the science of gene transfer and its clinical applications.

[64] Subhan MA, Filipczak N, Torchilin VP (2023). Advances with lipid-based nanosystems for siRNA delivery to breast cancers. Pharmaceuticals.

[65] Love KT, Mahon KP, Levins CG, Whitehead KA, Querbes W, Dorkin JR (2010). Lipid-like materials for low-dose, in vivo gene silencing. Proceedings of the National Academy of Sciences.

[66] Jain D, Prajapati SK, Jain A, Singhal R (2023). Nano-formulated siRNA-based therapeutic approaches for cancer therapy. Nano Trends.

[67] Sun R, Chen Y, Pei Y, Wang W, Zhu Z, Zheng Z (2024). The drug release of PLGA-based nanoparticles and their application in treatment of gastrointestinal cancers. Heliyon.

[68] Heo MB, Lim YT (2014). Programmed nanoparticles for combined immunomodulation, antigen presentation and tracking of immunotherapeutic cells. Biomaterials.

[69] Fève B, Bastard J-P (2009). The role of interleukins in insulin resistance and type 2 diabetes mellitus. Nature Reviews Endocrinology.

[70] Locksley RM, Killeen N, Lenardo MJ (2001). The TNF and TNF receptor superfamilies: integrating mammalian biology. Cell.

[71] Stuart LM, Ezekowitz RA (2008). Phagocytosis and comparative innate immunity: learning on the fly. Nature Reviews Immunology.

[72] Liu Y-J (2005). IPC: professional type 1 interferon-producing cells and plasmacytoid dendritic cell precursors. Annu Rev Immunol.

[73] Janeway Jr CA, Medzhitov R (2002). Innate immune recognition. Annual review of immunology.

[74] Amarante-Mendes GP, Adjemian S, Branco LM, Zanetti LC, Weinlich R, Bortoluci KR (2018). Pattern Recognition Receptors and the Host Cell Death Molecular Machinery. Frontiers in Immunology.

[75] Whitehead KA, Dahlman JE, Langer RS, Anderson DG (2011). Silencing or stimulation? siRNA delivery and the immune system. Annual review of chemical and biomolecular engineering.

[76] Kawai T, Akira S (2010). The role of pattern-recognition receptors in innate immunity: update on Toll-like receptors. Nature immunology.

[77] Bell JK, Askins J, Hall PR, Davies DR, Segal DM (2006). The dsRNA binding site of human Toll-like receptor 3. Proceedings of the National Academy of Sciences.

[78] Matsumoto M, Funami K, Tanabe M, Oshiumi H, Shingai M, Seto Y (2003). Subcellular localization of Toll-like receptor 3 in human dendritic cells. The Journal of Immunology.

[79] Applequist SE, Wallin RP, Ljunggren HG (2002). Variable expression of Toll-like receptor in murine innate and adaptive immune cell lines. International immunology.

[80] Bekeredjian-Ding IB, Wagner M, Hornung V, Giese T, Schnurr M, Endres S (2005). Plasmacytoid dendritic cells control TLR7 sensitivity of naive B cells via type I IFN. The Journal of Immunology.

[81] Kaushal A (2023). Innate immune regulations and various siRNA modalities. Drug Delivery and Translational Research.

[82] Forsbach A, Nemorin J-G, Montino C, Müller C, Samulowitz U, Vicari AP (2008). Identification of RNA sequence motifs stimulating sequence-specific TLR8-dependent immune responses. The Journal of Immunology.

[83] Gorden KB, Gorski KS, Gibson SJ, Kedl RM, Kieper WC, Qiu X (2005). Synthetic TLR agonists reveal functional differences between human TLR7 and TLR8. The Journal of Immunology.

[84] Zarember KA, Godowski PJ (2002). Tissue expression of human Toll-like receptors and differential regulation of Toll-like receptor mRNAs in leukocytes in response to microbes, their products, and cytokines. The journal of immunology.

[85] Meurs E, Chong K, Galabru J, Thomas NSB, Kerr IM, Williams BR (1990). Molecular cloning and characterization of the human double-stranded RNA-activated protein kinase induced by interferon. Cell.

[86] Zhang Z, Weinschenk T, Guo K, Schluesener HJ (2006). siRNA binding proteins of microglial cells: PKR is an unanticipated ligand. Journal of cellular biochemistry.

[87] Clemens MJ, ELIA A (1997). The double-stranded RNA-dependent protein kinase PKR: structure and function. Journal of interferon & cytokine research.

[88] Marques JT, Devosse T, Wang D, Zamanian-Daryoush M, Serbinowski P, Hartmann R (2006). A structural basis for discriminating between self and nonself double-stranded RNAs in mammalian cells. Nature biotechnology.

[89] Puthenveetil S, Whitby L, Ren J, Kelnar K, Krebs JF, Beal PA (2006). Controlling activation of the RNA-dependent protein kinase by siRNAs using site-specific chemical modification. Nucleic acids research.

[90] Williams BR (2001). Signal integration via PKR. Science's STKE.

[91] Kawai T, Akira S (2008). Toll-like receptor and RIG-1-like receptor signaling. Annals of the New York Academy of Sciences.

[92] Yoneyama M, Kikuchi M, Natsukawa T, Shinobu N, Imaizumi T, Miyagishi M (2004). The RNA helicase RIG-I has an essential function in double-stranded RNA-induced innate antiviral responses. Nature immunology.

[93] Conde J, Arnold CE, Tian F, Artzi N (2016). RNAi nanomaterials targeting immune cells as an anti-tumor therapy: the missing link in cancer treatment?. Materials Today.

[94] Yusuf A, Almotairy ARZ, Henidi H, Alshehri OY, Aldughaim MS (2023). Nanoparticles as drug delivery systems: a review of the implication of nanoparticles' physicochemical properties on responses in biological systems. Polymers.

[95] Conde J, Ambrosone A, Hernandez Y, Tian F, McCully M, Berry CC (2015). 15 years on siRNA delivery: Beyond the State-of-the-Art on inorganic nanoparticles for RNAi therapeutics. Nano Today.

[96] Zhang M, Gao S, Yang D, Fang Y, Lin X, Jin X (2021). Influencing factors and strategies of enhancing nanoparticles into tumors in vivo. Acta pharmaceutica Sinica B.

[97] Gao S, Dagnaes-Hansen F, Nielsen EJB, Wengel J, Besenbacher F, Howard KA (2009). The Effect of Chemical Modification and Nanoparticle Formulation on Stability and Biodistribution of siRNA in Mice. Molecular Therapy.

[98] Zuckerman JE, Choi CHJ, Han H, Davis ME (2012). Polycation-siRNA nanoparticles can disassemble at the kidney glomerular basement membrane. Proceedings of the National Academy of Sciences.

[99] Kumari N, Siddhanta K, Panja S, Joshi V, Jogdeo C, Kapoor E (2023). Oral Delivery of Nucleic Acid Therapies for Local and Systemic Action. Pharmaceutical research.

[100] Whitehead KA, Langer R, Anderson DG (2009). Knocking down barriers: advances in siRNA delivery. Nature Reviews Drug Discovery.

[101] Rabinovich GA, Gabrilovich D, Sotomayor EM (2007). Immunosuppressive Strategies that are Mediated by Tumor Cells. Annual Review of Immunology.

[102] Sajid MI, Moazzam M, Kato S, Yeseom Cho K, Tiwari RK (2020). Overcoming Barriers for siRNA Therapeutics: From Bench to Bedside. Pharmaceuticals (Basel, Switzerland).

[103] Conner SD, Schmid SL (2003). Regulated portals of entry into the cell. Nature.

[104] Amoozgar Z, Goldberg MS (2015). Targeting myeloid cells using nanoparticles to improve cancer immunotherapy. Advanced drug delivery reviews.

[105] Shi SJ, Wang LJ, Han DH, Wu JH, Jiao D, Zhang KL (2019). Therapeutic effects of human monoclonal PSMA antibody-mediated TRIM24 siRNA delivery in PSMA-positive castration-resistant prostate cancer. Theranostics.

[106] França A, Aggarwal P, Barsov EV, Kozlov SV, Dobrovolskaia MA, González-Fernández Á (2011). Macrophage scavenger receptor A mediates the uptake of gold colloids by macrophages in vitro. Nanomedicine.

[107] Conde J, Bao C, Tan Y, Cui D, Edelman ER, Azevedo HS (2015). Dual targeted immunotherapy via in vivo delivery of biohybrid RNAi-peptide nanoparticles to tumor-associated macrophages and cancer cells. Advanced functional materials.

[108] Barua S, Mitragotri S (2014). Challenges associated with Penetration of Nanoparticles across Cell and Tissue Barriers: A Review of Current Status and Future Prospects. Nano Today.

[109] Guo S, Huang L (2011). Nanoparticles Escaping RES and Endosome: Challenges for siRNA Delivery for Cancer Therapy. Journal of Nanomaterials.

[110] Oliveira S, van Rooy I, Kranenburg O, Storm G, Schiffelers RM (2007). Fusogenic peptides enhance endosomal escape improving siRNA-induced silencing of oncogenes. International journal of pharmaceutics.

[111] Akita H, Kogure K, Moriguchi R, Nakamura Y, Higashi T, Nakamura T (2010). Nanoparticles for ex vivo siRNA delivery to dendritic cells for cancer vaccines: Programmed endosomal escape and dissociation. Journal of Controlled Release.

[112] Moazzam M, Zhang M, Hussain A, Yu X, Huang J, Huang Y (2024). The landscape of nanoparticle-based siRNA delivery and therapeutic development. Molecular Therapy.

[113] Ali Zaidi SS, Fatima F, Ali Zaidi SA, Zhou D, Deng W, Liu S (2023). Engineering siRNA therapeutics: challenges and strategies. Journal of Nanobiotechnology.

[114] Yu SS, Lau CM, Barham WJ, Onishko HM, Nelson CE, Li H (2013). Macrophage-specific RNA interference targeting via “click”, mannosylated polymeric micelles. Molecular pharmaceutics.

[115] Alexis F, Pridgen E, Molnar LK, Farokhzad OC (2008). Factors Affecting the Clearance and Biodistribution of Polymeric Nanoparticles. Molecular Pharmaceutics.

[116] Thiele L, Merkle HP, Walter E (2003). Phagocytosis and Phagosomal Fate of Surface-Modified Microparticles in Dendritic Cells and Macrophages. Pharmaceutical Research.

[117] Lu C, Liu Y, Ali NM, Zhang B, Cui X (2022). The role of innate immune cells in the tumor microenvironment and research progress in anti-tumor therapy. Frontiers in immunology.

[118] Yang M, Li J, Gu P, Fan X (2021). The application of nanoparticles in cancer immunotherapy: Targeting tumor microenvironment. Bioactive materials.

[119] Leuschner F, Dutta P, Gorbatov R, Novobrantseva TI, Donahoe JS, Courties G (2011). Therapeutic siRNA silencing in inflammatory monocytes in mice. Nature Biotechnology.

[120] Hobo W, Novobrantseva TI, Fredrix H, Wong J, Milstein S, Epstein-Barash H (2013). Improving dendritic cell vaccine immunogenicity by silencing PD-1 ligands using siRNA-lipid nanoparticles combined with antigen mRNA electroporation. Cancer Immunology, Immunotherapy.

[121] Warashina S, Nakamura T, Harashima H (2011). A20 silencing by lipid envelope-type nanoparticles enhances the efficiency of lipopolysaccharide-activated dendritic cells. Biological & pharmaceutical bulletin.

[122] Alshamsan A, Hamdy S, Haddadi A, Samuel J, El-Kadi AOS, Uludağ H (2011). STAT3 Knockdown in B16 Melanoma by siRNA Lipopolyplexes Induces Bystander Immune Response In Vitro and In Vivo. Translational Oncology.

[123] Alshamsan A, Haddadi A, Hamdy S, Samuel J, El-Kadi AOS, Uludağ H (2010). STAT3 Silencing in Dendritic Cells by siRNA Polyplexes Encapsulated in PLGA Nanoparticles for the Modulation of Anticancer Immune Response. Molecular Pharmaceutics.

[124] Rich JN (2007). Cancer stem cells in radiation resistance. Cancer research.

[125] Xu L, Shen W, Wang B, Wang X, Liu G, Tao Y (2016). Efficient siRNA delivery using PEG-conjugated PAMAM dendrimers targeting vascular endothelial growth factor in a CoCl2-induced neovascularization model in retinal endothelial cells. Current drug delivery.

[126] Nam J-P, Nam K, Jung S, Nah J-W, Kim SW (2015). Evaluation of dendrimer type bio-reducible polymer as a siRNA delivery carrier for cancer therapy. Journal of Controlled Release.

[127] Vesuna F, Lisok A, Kimble B, Raman V (2009). Twist modulates breast cancer stem cells by transcriptional regulation of CD24 expression. Neoplasia.

[128] Finlay J, Roberts CM, Dong J, Zink JI, Tamanoi F, Glackin CA (2015). Mesoporous silica nanoparticle delivery of chemically modified siRNA against TWIST1 leads to reduced tumor burden. Nanomedicine: Nanotechnology, Biology and Medicine.

[129] Cho H, Yeh E-C, Sinha R, Laurence TA, Bearinger JP, Lee LP (2012). Single-step nanoplasmonic VEGF165 aptasensor for early cancer diagnosis. ACS nano.

[130] Engelberth SA, Hempel N, Bergkvist M (2015). Chemically modified dendritic starch: a novel nanomaterial for siRNA delivery. Bioconjugate chemistry.

[131] Lee JB, Zhang K, Tam YYC, Quick J, Tam YK, Lin PJC (2016). A Glu-urea-Lys Ligand-conjugated Lipid Nanoparticle/siRNA System Inhibits Androgen Receptor Expression In Vivo. Molecular Therapy - Nucleic Acids.

[132] Taratula O, Kuzmov A, Shah M, Garbuzenko OB, Minko T (2013). Nanostructured lipid carriers as multifunctional nanomedicine platform for pulmonary co-delivery of anticancer drugs and siRNA. Journal of Controlled Release.

[133] Kim SI, Shin D, Choi TH, Lee JC, Cheon G-J, Kim K-Y (2007). Systemic and Specific Delivery of Small Interfering RNAs to the Liver Mediated by Apolipoprotein A-I. Molecular Therapy.

[134] Lu C, Stewart DJ, Lee JJ, Ji L, Ramesh R, Jayachandran G (2012). Phase I clinical trial of systemically administered TUSC2 (FUS1)-nanoparticles mediating functional gene transfer in humans. PloS one.

[135] Muralidharan R, Babu A, Amreddy N, Basalingappa K, Mehta M, Chen A (2016). Folate receptor-targeted nanoparticle delivery of HuR-RNAi suppresses lung cancer cell proliferation and migration. Journal of nanobiotechnology.

[136] Xia W, Li Y, Lou B, Wang P, Gao X, Lin C (2013). Bioreducible PEI-siRNA nanocomplex for liver cancer therapy: transfection, biodistribution, and tumor growth inhibition in vivo. Journal of Nanomaterials. 2013.

[137] Ertas IE, Gulcan M, Bulut A, Yurderi M, Zahmakiran M (2016). Metal-organic framework (MIL-101) stabilized ruthenium nanoparticles: Highly efficient catalytic material in the phenol hydrogenation. Microporous and Mesoporous Materials.

[138] Chen Y, Xu M, Guo Y, Tu K, Wu W, Wang J (2016). Targeted chimera delivery to ovarian cancer cells by heterogeneous gold magnetic nanoparticle. Nanotechnology.

[139] Tian J, Chen J, Ge C, Liu X, He J, Ni P (2016). Synthesis of PEGylated ferrocene nanoconjugates as the radiosensitizer of cancer cells. Bioconjugate chemistry.

[140] Saad M, Garbuzenko OB, Minko T Co-delivery of siRNA and an anticancer drug for treatment of multidrug-resistant cancer. 2008; 3: 761-76.

[141] Li W, Wang Y, Zhao H, Zhang H, Xu Y, Wang S (2019). Identification and transcriptome analysis of erythroblastic island macrophages. Blood, The Journal of the American Society of Hematology.

[142] Finlay J, Roberts CM, Lowe G, Loeza J, Rossi JJ, Glackin CA (2015). RNA-based TWIST1 inhibition via dendrimer complex to reduce breast cancer cell metastasis. BioMed research international.

[143] Liu X, Zhou J, Yu T, Chen C, Cheng Q, Sengupta K (2014). Adaptive amphiphilic dendrimer-based nanoassemblies as robust and versatile siRNA delivery systems. Angewandte Chemie International Edition.

[144] Wang H, Zhang J, Yu H (2007). Elemental selenium at nano size possesses lower toxicity without compromising the fundamental effect on selenoenzymes: comparison with selenomethionine in mice. Free Radical Biology and Medicine.

[145] Levina A, Mitra A, Lay PA (2009). Recent developments in ruthenium anticancer drugs. Metallomics.

[146] Bhattacharjee R, Jana A, Nandi A, Sinha A, Bhattacharjee A, Mitra S (2022). Synergy of nanocarriers with CRISPR-Cas9 in an emerging technology platform for biomedical appliances: Current insights and perspectives. Materials & Design.

